# Bidirectional Interactions Between Immune Regulation and the Insulin-like Growth Factor Axis in Colorectal Cancer

**DOI:** 10.3390/ijms27083666

**Published:** 2026-04-20

**Authors:** Hilmaris Centeno-Girona, Sheila N. López-Acevedo, Camille Zenón-Meléndez, Olga L. Díaz-Miranda, Elba V. Caraballo

**Affiliations:** 1 Division of Shared Resources and Scientific Operations, University of Puerto Rico Comprehensive Cancer Center, San Juan 00936, Puerto Rico; hcenteno@cccupr.org (H.C.-G.); shelopez@cccupr.org (S.N.L.-A.); czenon@cccupr.org (C.Z.-M.); oldiaz@cccupr.org (O.L.D.-M.); 2 Division of Clinical and Translational Cancer Research, University of Puerto Rico Comprehensive Cancer Center, San Juan 00936, Puerto Rico

**Keywords:** insulin-like growth factor, colorectal cancer, tumor microenvironment, immune regulation

## Abstract

Colorectal cancer (CRC) is the third most commonly diagnosed malignancy worldwide, with molecular heterogeneity complicating early detection and treatment stratification. The insulin-like growth factor (IGF) axis interacts bidirectionally with immune regulatory mechanisms in ways that shape tumor phenotype and therapeutic vulnerability. This review synthesizes evidence on how IGF signaling orchestrates immunosuppression through effects on tumor-associated macrophages, regulatory T cells, and myeloid-derived suppressor cells, while inflammatory cytokines reciprocally modulate IGF bioavailability. Three mechanistic principles emerge: IGF binding protein 2 (IGFBP-2) functions as a central coordinator linking growth factor signaling to immune evasion through STAT3-dependent pathways driving M2 macrophage polarization and regulatory T cell differentiation; IGF–immune crosstalk varies considerably across molecular subtypes, with microsatellite-stable tumors exhibiting high reliance on IGF-I receptor-mediated immune silencing; and local paracrine IGF production increasingly dominates over systemic regulation as disease progresses. These bidirectional connections establish self-reinforcing circuits that determine whether tumors remain immunologically responsive or develop immune exclusion. Multi-marker panels incorporating IGFBP-2 alongside complementary biomarkers have shown improved diagnostic performances for early CRC detection, underscoring the need for the large-scale prospective clinical evaluation of IGF network components as biomarkers for CRC in diverse populations. The convergence of IGF signaling with checkpoint regulation suggests that combined targeting warrants investigation for resistance in tumors lacking effective immunotherapy options.

## 1. Introduction

### 1.1. Colorectal Cancer and the Need for Novel Biomarker Strategies

#### 1.1.1. Global Incidence and Mortality

Globally, colorectal cancer (CRC) is the third most commonly diagnosed type of cancer, accounting for approximately 10% of all cancer cases. It is also the second leading cause of cancer-related deaths worldwide, with over 1.9 million new cases and approximately 904,000 deaths reported in 2022 [1]. CRC occurs about four times more frequently in high-income countries compared to low- and middle-income countries, while mortality rates are roughly twice as high in high-income countries relative to low- and middle-income countries [2].

#### 1.1.2. Molecular Heterogeneity

CRC exhibits remarkable molecular heterogeneity between patients, due to variations in tumor origin, genetic background, and environmental exposures, as well as within individual tumors, where genetically and epigenetically distinct cellular populations coexist and interact with the microenvironment. Additionally, metastatic lesions can differ from one another and evolve, further complicating treatment [3,4]. To navigate this complexity, molecular subtyping has been developed as a strategy for classifying tumors based on genomic and transcriptomic biomarkers, providing deeper insights into the signaling pathways that drive tumor initiation, progression, and therapeutic response [5]. Microsatellite instability (MSI) represents one of the earliest subtyping criteria with established clinical utility for CRC [5,6]. MSI arises from defective DNA mismatch repair (MMR), typically caused by inactivation of MMR genes such as MLH1, PMS2, MSH2, or MSH6 [7]. Tumors are classified as MSI-High (MSI-H), MSI-Low (MSI-L), or microsatellite-stable (MSS), with MSI-L and MSS often analyzed together due to their similar biological and clinical features [8]. MSI-H characterizes approximately 15% of CRCs, resulting from either germline mutations in MMR genes associated with Lynch Syndrome (~3%) or somatic alterations that lead to sporadic MSI-H (~12%). In contrast, the remaining 85% of CRCs display MSS or MSI-L and typically develop through chromosomal instability-driven pathways [9]. Although genome-based molecular typing represents a key dimension of CRC heterogeneity, advances in transcriptomic profiling over the past decades have led to the development of various molecular classification frameworks [5]. Among these, the Consensus Molecular Subtype (CMS) classification identifies four principal subtypes with distinct mutational, transcriptomic, and clinical features: CMS1 (immune-infiltrated), CMS2 (canonical), CMS3 (metabolic), and CMS4 (mesenchymal/stromal-rich) [10,11].

#### 1.1.3. Limitations in Current Early Detection Approaches

Early detection of CRC remains challenging due to the asymptomatic nature of early disease and suboptimal screening adherence. Despite established guidelines, screening adherence ranges from 19% to 69% across Europe, approximates 55% in Canada, and is roughly 50% in the United States [12]. Furthermore, early manifestations are often confused with other inflammatory conditions, such as colitis, complicating timely diagnosis [13]. Currently, early CRC detection primarily relies on stool-based tests, endoscopic procedures, and imaging techniques, each with inherent limitations, including low sensitivity for precancerous lesions, high cost, invasiveness, patient discomfort, and limited population acceptance [13]. Moreover, the rising incidence of early-onset CRC, defined as diagnosis before age 50, further exposes gaps in screening paradigms historically designed for older adults [14].

Advances in omics technologies have enabled the identification of genomic, transcriptomic, proteomic, and metabolomic biomarkers as potential tools for early CRC detection. However, most mechanisms and molecules involved are still under investigation, and only a limited number have demonstrated clinical relevance, highlighting the need for further translation from research to practical diagnostic applications [13]. Liquid biopsy has emerged as a minimally invasive method for detecting these markers, although low concentrations of circulating tumor-derived material and technical challenges continue to limit its clinical implementation [13].

### 1.2. The IGF Axis: A Brief Overview

The insulin-like growth factor (IGF) axis comprises a tightly regulated network that coordinates cellular growth, differentiation, metabolism, and tissue homeostasis across multiple organ systems. Through dynamic connection between ligands, soluble binding proteins, and receptor complexes, this network responds to extracellular stimuli to activate intracellular signaling cascades to regulate coordinated cellular processes. These regulatory mechanisms ensure appropriate tissue responses, while dysregulation can contribute to pathological states, including cancer, metabolic disorders, and defective tissue repair [15,16].

#### 1.2.1. Key Components: Ligands, Receptors, Binding Proteins

This axis is composed of ligands (IGF-I, IGF-II, and insulin) that bind to structurally related cell surface receptors: IGF receptors 1 and 2 (IGF-1R and -2R) and insulin receptors A and B (IR-A and -B) [17,18]. Each receptor can dimerize with an identical partner to form homodimers or with a different receptor to form hybrid heterodimers, thereby modulating ligand specificity and downstream signaling [19,20]. The human IR exists as two splice variants: IR-A, which binds both insulin and IGF-II with high affinity and preferentially activates mitogenic pathways, and IR-B, which primarily mediates metabolic function [16,17,21]. IR-A is frequently overexpressed in cancer, including CRC, where IGF-II/IR-A signaling contributes to tumor progression [22,23]. IGF-1R mediates potent mitogenic signaling through high-affinity binding to IGF-I and IGF-II, while IGF-2R acts as a scavenger receptor, binding, internalizing, and targeting IGF-II for lysosomal degradation, thereby limiting excessive proliferative signaling [17]. Upon receptor activation, these ligands engage major intracellular signaling cascades, including the phosphatidylinositol 3-kinase (PI3K)/protein kinase B (also known as AKT) and oncogenic rat sarcoma (RAS)/rapidly accelerated fibrosarcoma (RAF)/mitogen-activated protein kinase (MAPK) pathways, which drive metabolic and mitogenic responses [15,24]. The availabilities of IGF-I and -II are further modulated by six high-affinity IGF binding proteins (IGFBP-1–6), which form IGF/IGFBP complexes that protect the ligands from degradation, prolong their half-lives, influence their localization within tissues, and regulate their binding with receptors [15,17].

#### 1.2.2. Downstream Signaling Pathways

**Figure 1:**
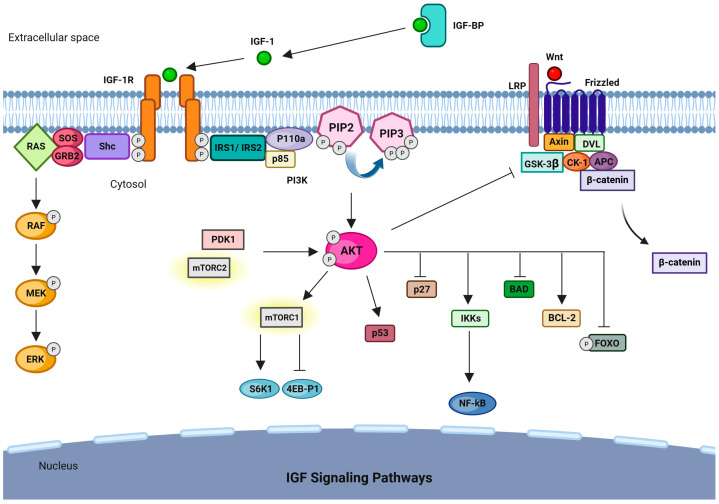
The IGF signaling pathways in colorectal cancer. In the extracellular space, IGFBPs sequester IGF-1, limiting receptor access. IGF-I is released through IGFBP proteolysis by MMPs and pappalysins, or through reduced binding affinity, enabling ligand receptor engagement. This figure illustrates the canonical intracellular signaling cascades activated upon IGF-1 binding to IGF-1R in colorectal epithelial cells. Ligand binding recruits adaptor proteins (Shc, IRS1/2), activating two principal downstream pathways: (1) RAS/RAF/MEK/ERK signaling promoting proliferation and differentiation, and (2) PI3K/AKT/mTOR signaling regulating survival, metabolism, and protein synthesis. AKT phosphorylation inhibits proapoptotic factors (BAD, FOXO, p27) while activating NF-κB and mTORC1. Crosstalk with Wnt/β-catenin signaling is shown. Phosphorylated AKT-mediated phosphorylation inhibits GSK-3β, preventing β-catenin degradation and enabling nuclear translocation to activate Wnt target genes. Created in BioRender. Zenón, C. (2026) https://BioRender.com/r4vynu6, accessed on 20 March 2026.

Activation of IGF-1R initiates the PI3K/AKT and MAPK/ERK signaling cascades (Figure 1) [18,25,26]. IGF-I binding recruits adaptor proteins such as Src homology 2 domain containing (Shc) and insulin receptor substrate 1 and 2 (IRS1 and IRS2), activating the Ras/RAF/MEK/ERK signaling and PI3K-mediated AKT pathways [16]. The PI3K/AKT pathway controls cellular responses such as proliferation, differentiation, apoptosis, and migration, in part through the activation of mammalian target of rapamycin (mTOR) complexes (mTORC1 and mTORC2) or inhibition of the Forkheadbox subclass O family members FOXO1, FOXO3, and FOXO4. Furthermore, AKT targets the proapoptotic Bcl-2 family member BAD [27,28], modulating cell cycle regulators such as p27 [21]. Additionally, AKT-mediated phosphorylation of glycogen synthase kinase 3 (GSK3) beta inactivates this kinase, preventing β-catenin degradation by the destruction complex and enabling its nuclear translocation to activate Wnt target genes [29,30,31]. This crosstalk between PI3K/AKT and Wnt/β-catenin signaling is particularly relevant in CRC, where Wnt pathway dysregulation is a frequent oncogenic driver [32]. Deregulation of the PI3K/Akt/mTOR pathway drives CRC progression and contributes to drug resistance [32,33].

Beyond their direct effects on tumor cells, the PI3K/AKT/mTOR and MAPK/ERK pathways activated by IGF-1R also play a role in shaping the immune environment in CRC [34]. The PI3K/AKT pathway promotes the polarization of macrophages toward an immunosuppressive M2 phenotype and supports the expansion of regulatory T cells through mTOR signaling [35,36,37]. Simultaneously, MAPK/ERK signaling, together with NF-κB activated downstream of AKT, enhances the immunosuppressive activity of MDSCs by increasing arginase-1, iNOS, and reactive oxygen species [38,39,40]. In addition, multiple signaling pathways, including STAT3, HIF-1α, NF-κB, and ERK, converge at the PD-L1 promoter, linking IGF-1R activation to immune checkpoint evasion [41].

#### 1.2.3. IGF/IGFR/IGFBP Expression and Clinical Relevance in CRC

Clinical and translational evidence supports the relevance of IGF axis dysregulation in CRC. IGF-1R is frequently overexpressed in CRC tissues compared to normal mucosa and has been associated with advanced stage and invasive behavior, although its prognostic significance varies across cohorts [42,43,44,45]. IGF-1R activation is also critical for the development of anti-EGFR therapy resistance, although little is known about its role in the immune system in this context [46]. Beyond receptor overexpression, alterations in IGF ligands further contribute to pathway activation. IGF-II is also commonly overproduced in CRC, often due to loss of imprinting, leading to increased autocrine and paracrine signaling via IGF-1R and IR-A, thereby promoting tumor growth and invasion and contributing to adverse clinical outcomes [47].

Moreover, circulating components of the IGF system show prognostic relevance. Plasma IGFBP-2 levels are significantly elevated in CRC patients, correlating with the tumor burden and stage, and independently predicting poorer overall survival, supporting its potential as a diagnostic and prognostic biomarker [48]. In contrast, IGFBP-6 exhibits tumor-suppressive properties, with reduced expression in CRC tissues associated with enhanced proliferation, invasion, and metastatic potential and unfavorable survival [49]. In addition to extracellular and circulating factors, regulation of IGF signaling also occurs at the post-transcriptional level. In this context, the IGF-II mRNA-binding protein (IGF2BP)-1 is frequently overexpressed in CRC and is an independent prognostic factor associated with aggressive tumor behavior and therapy resistance [50,51]. IGFBP-3 presents a distinct profile from that of IGFBP-2 and -6, exhibiting a complex yet therapeutically promising function, as it can be leveraged as a natural IGF antagonist to sequester oncogenic ligands without interfering with IGF signaling, thereby minimizing metabolic toxicities [52]. Serum IGFBP 3 levels are often lower in CRC patients compared with healthy controls, and this drop correlates with the tumor stage, size, and differentiation [53]. Low circulating IGFBP 3 has been associated with worse prognoses and survival in metastatic CRC cohorts, likely because it allows higher IGF I and IGF II to drive unrestrained PI3K/AKT and MAPK signaling [24,53]. At the tissue level, IGFBP 3 can be proapoptotic and antiproliferative in CRC cells, and its local expression inversely correlates with adenoma risk [54,55]. Interestingly, IGFBP 3 is also subject to abnormal glycosylation in CRC, which impairs its binding capacity and may contribute to the aggressive phenotype [56]. Clinically, serum IGFBP 3 can act as a complementary biomarker to IGFBP 2 as a potential targeted therapy [29,57]. Overall, these findings underscore the clinical relevance of IGF axis dysregulation in CRC and highlight multiple components of this pathway as promising biomarkers and therapeutic targets.

#### 1.2.4. Post-Transcriptional Regulation of IGF Components by Non-Coding RNAs (ncRNAs)

Beyond transcriptional and epigenetic mechanisms, non-coding RNAs (ncRNAs) constitute an additional regulatory layer that modulates IGF axis component expression in CRC with emerging implications for immune regulation [58]. MicroRNAs (miRNAs) function as post-transcriptional repressors by binding to the 3′-untranslated region (3′-UTR) of target mRNAs, and several have been identified as direct regulators of the IGF-1R cascade in CRC. miR-497 targets the 3′-UTR of insulin receptor substrate-1 (IRS-1), and its downregulation in CRC tissues has been correlated with IRS-1 overexpression and enhanced proliferative signaling [59,60], while the miR-143/145 cluster, a well-characterized tumor suppressor frequently silenced in CRC, directly represses IGF-1R expression [61]. miR-223 is another highly conserved miRNA that regulates metabolism, immune response, and inflammation and suppresses the IGF-1R/PI3K/Akt pathway [62]. Additional miRNAs regulate IGF-II availability at the ligand level, including miR-483, miR-486-5p, and miR-491-5p, which collectively modulate IGF-II-dependent proliferation and invasion pathways in CRC [60,63,64]. Circular RNAs (circRNAs) function as miRNA sponges that reactivate IGF signaling. Notable circRNAs include circHIPK3, which sequesters miR-7 to relieve its suppressive effect on IGF-1R, thereby promoting tumor proliferation and metastasis through PI3K/AKT pathway reactivation [65,66], and circRUNX1, which upregulates IGF-1 expression by sequestering miR-145-5p [67].

Long non-coding RNA (lncRNA) also participate in IGF axis regulation. The lncRNA H19, encoded within the imprinted IGF-II locus, is upregulated in CRC and exerts oncogenic functions, including modulating the IGF system by acting as a molecular decoy [29,63]. CRNDE has contributed to IGF signaling-driven metabolic reprogramming associated with aerobic glycolysis [29,68]. Additional lncRNAs with established roles in CRC, NEAT1 and MALAT1, promote colorectal cancer progression by activating AKT signaling pathways, with MALAT1 also contributing to immune regulation through PD-L1 modulation [69,70,71].

IGF2BPs 1–3 function as post-transcriptional regulators of target mRNA stability and translation. IGF2BP1 is an independent prognostic factor associated with aggressive CRC behavior and chemotherapy resistance [29], while IGF2BP3 stabilizes EGFR mRNA via N6-methyladenosine (m6A) modification, mediating cetuximab resistance [72]. The m6A modification landscape adds another layer of complexity to this post-transcriptional regulatory network. METTL3-dependent m6A can stabilize IGF2BP3 target mRNAs contributing to cetuximab resistance, and more broadly, increased METTL3 activity promotes CRC progression by activating the AKT/mTOR and MAPK pathways [72,73,74,75]. In parallel, METTL14-mediated m6A modification has been linked to increased PD-L1 expression and immune evasion in CRC, directly connecting this regulatory layer to immune checkpoint control [76]. While some miRNAs silence gene expression, the X inactive-specific transcript (XIST) lncRNA similarly modulates CRC proliferation and metastasis by sequestering miR-200b [77,78]. This oncogenic activity is primarily regulated by METTL14, which acts by inducing XIST degradation. The conserved structure of these ncRNAs-IGF regulatory networks across cancer types is well supported by extensive studies on breast cancer, where miRNAs, circRNAs, and lncRNAs targeting IGF-1R, IRS-1, and IGF-II have been systematically characterized [79]. Together, these findings strengthen the biological possibility and translational relevance of similar regulatory mechanisms in CRC. Through these ncRNA-mediated mechanisms, dysregulation of IGF-1R and its downstream PI3K/AKT/mTOR signaling drives immunosuppressive changes in CRC, including increased recruitment of MDSCs, expansion of Tregs, and stabilization of PD-L1 [58,80,81,82]. Together, these effects directly link post-transcriptional alterations of IGF signaling mediators to immune evasion. The immune implications of these ncRNA-IGF circuits are discussed in Section 3.1.3 and Section 3.2.1. The key components of the IGF axis and their roles in CRC immunity are summarized in Table 1.

### 1.3. Immune Contexture in CRC

CRC develops within a dynamic immune microenvironment where the composition, spatial distribution, and functional state of immune cell populations, collectively termed the immune contexture, serve as clinical predictors of patient outcome and therapeutic response [113,114,115,116]. The CRC tumor microenvironment (TME) harbors important immune populations, including tumor-infiltrating lymphocytes (TILs), tumor-associated macrophages (TAMs), myeloid-derived suppressor cells (MDSCs) and regulatory T cells (Tregs). These cell types shape the immunosuppressive CRC TME, influencing tumor progression, immune evasion, and therapy responses [117]. TIL infiltration is associated with favorable outcomes, though high infiltration is less common in MSS CRC in compared to MSI-H [118]. Meanwhile, TAMs, MDSCs, and Tregs collectively establish immunosuppressive conditions through cytokine secretion, effector cell inhibition, and vascular remodeling [119,120,121,122,123,124,125,126]. These populations collectively determine whether CRC tumors develop immunologically “hot” phenotypes, characterized by effector T cell infiltration and antigen presentation typical of MSI-H tumors, or “cold” phenotypes, with immune exclusion and suppression characteristic of MSS CRC [121,125,127,128].

The complex immune landscape in CRC intersects with growth factor signaling pathways, particularly with the IGF network. Recent studies reveal that this intersection is fundamentally bidirectional. IGF network components actively suppress immune surveillance by degrading immune stimuli, blocking antitumor lymphocyte infiltration and promoting immunosuppressive cell phenotypes. Reciprocally, inflammatory mediators, including interleukins and chemokines, upregulate IGF-1R and IGFBP expression, establishing feed-forward loops that sustain immune evasion and proliferation [129]. This review synthesizes the current evidence on the bidirectional interactions between the IGF/IGFR/IGFBP signaling pathway and the immunoregulatory mechanisms that underlie CRC development and progression, with implications for combined IGF–immune signatures as potential biomarkers for early CRC detection.

## 2. Immune Regulation of IGF Axis

### 2.1. Cytokine-Mediated Modulation of IGF Signaling

The IGF pathway is continually modulated by inflammatory factors within the TME, in which cytokines secreted by immune and stromal cells affect the synthesis of IGFs, IGFRs, and IGFBPs. The presence of a prominent inflammatory component within CRC, especially in advanced stages, suggests that the regulation of the IGF pathway by cytokines is a significant but often ignored aspect of this important growth factor cascade [22]. Cytokines control the IGF network through altered intracellular signaling as well as transcriptional and post-transcriptional mechanisms. The influence of pro- and anti-inflammatory cytokine signaling on the IGF availability and IGFR signaling capacity are summarized below.

#### 2.1.1. Pro-Inflammatory Cytokines

##### IL-6 Effects on IGF-I and IGFBP Expression

Among pro-inflammatory cytokines, IL-6 has the strongest association with regulation of IGF signaling [130,131]. During general chronic inflammation, IL-6 works through the janus kinase (JAK)/STAT3 pathway to inhibit the production of IGF-I by inducing suppressor cytokine signaling 3 (SOCS3) [131]. IL-6 also increases circulating IGFBP-1 and -6, potentially decreasing the systemic free IGF availability and relocating energy towards immune defense, a mechanism demonstrated in teleost fish models and general mammalian immune responses [130,132]. However, in CRC, the relationship is more complex. Chronically elevated levels of IL-6 and IL-11 have been shown to sustain STAT3 activation in tumor cells and cancer-associated fibroblasts (CAFs), which can enhance IGF-1R expression and downstream signaling sensitivity, even in the context of reduced systemic IGF-I availability [129,133]. Moreover, local effects differ from systemic regulation, as IL-1β coordinates with IL-6 to reduce the secretion of IGFBP-2 and -4 in CRC epithelial cells, paradoxically increasing the local IGF availability and promoting tumor cell proliferation [108].

##### TNF-α-Mediated Suppression of IGF-I Production and IGFBP Dysregulation

Tumor necrosis factor-alpha (TNF-α) signaling is also interconnected with the IGF axis. TNF-α reduces IGF-I gene expression in hepatocytes, the primary source of circulating IGF-I [84,130]. However, TNF-α affects IGF-I signaling through additional mechanisms, including changes in IGFBP expression and the increased proteolysis of IGFBP-3 and IGFBP-4, which can briefly elevate free IGF levels but may ultimately decrease IGF availability under sustained inflammatory conditions, mechanisms characterized in general physiological models [134]. In CRC, IGF-1R signaling protects cells from TNF-α-induced apoptosis via a nuclear factor kappa B (NF-κB)-dependent mechanism. While TNF-α alone can induce apoptosis, the presence of IGF-1R signaling prevents this cytokine-induced cell death by upregulating anti-apoptotic proteins, including Survivin and Bcl-xL [135,136]. These findings suggest that although inflammation suppresses systemic IGF-I production, tumor-intrinsic mechanisms such as NF-κB activation, increased IGF-1R expression, and IGFBP remodeling can sustain or even enhance IGF-1R signaling locally within the TME across various malignancies, including CRC [129,137].

##### IFN-γ-Mediated Suppression of IGF-1R Signaling

Interferon gamma (IFN-γ), a cytokine with a connection to Th1-polarized immune responses, is a negative regulator of IGF signaling [127,138]. IFN-γ has been shown to induce growth arrest in epithelial and malignant cells in part by opposing the PI3K/AKT signaling pathway, a mechanism documented in ovarian and pancreatic cancer [138]. This form of negative regulation may be especially important in immunogenic CRC subtypes, like MSI-H tumors, where the role of IFN-γ signaling is well established in an antitumor immune context [127].

##### Impacts of IL-1β Inflammatory Cascade

IL-1β is a strong inducer of the inflammatory cascade and has been prominently implicated in chronic tissue inflammation and a tumor-permissive environment in CRC [139,140]. The IL-1β signal transduction pathway activates the expression of matrix metalloproteinases (MMPs) and proteolytic enzymes [141,142], leading to the breakdown of the extracellular matrix (ECM) components and the cleavage of IGFBPs in various malignancies, including CRC [52,143]. This proteolytic environment augments the local concentration of free IGF-I, even when systemic IGF-I levels are reduced. This paracrine modulation of IGF is particularly relevant in chronic inflammation in colorectal tumors, where IL-1β promotes tissue remodeling, angiogenesis, and invasiveness [32,144], thereby promoting tumor progression through locally sustained pro-survival signaling [108].

#### 2.1.2. Anti-Inflammatory Mediators

##### Indirect Effects of IL-10 on IGF Axis

IL-10, a major anti-inflammatory cytokine secreted by Tregs, macrophages, and dendritic cells, suppresses pro-inflammatory cytokine production by activated macrophages, dendritic cells in the TME, while also inhibiting effector cell function (CD4+, Th17, CD8+ cytotoxic T cells) [145,146,147,148]. Mechanistically, IL-10 activates the JAK1/tyrosine kinase 2(TYK2)/STAT3 signaling pathway; this activation transcriptionally represses pro-inflammatory genes and inhibits NF-κB-driven cytokines like IL-6 and TNF-α [147,149,150]. In addition to its conventional role, IL-10 indirectly modulates IGF bioavailability. For instance, IGF-I has been found to promote IL-10 secretion in T cells and mononuclear cells, as shown in prostate cancer and autoimmune models [34,93,151], while IL-10 secretion reciprocally influences IGF availability by reducing levels of TNF-α and IL-1β, demonstrated in skeletal myoblasts [152], which drive IGFBP expression and proteolysis [108,153]. IL-10 also promotes M2 macrophage polarization; these M2 macrophages release MMP-9, which cleaves IGFBPs to increase IGF bioavailability, and secrete TGF-β, which stimulates IGF-II production by CAFs [23,90,154,155]. M2 additionally secretes IGF-I and -II directly to the TME, establishing autocrine and paracrine loops that sustain tumor growth, as demonstrated in thyroid cancer, breast cancer, and immunometabolic models [23,86,156,157].

##### TGF-β’s Context-Dependent Modulation of IGF

Transforming growth factor (TGF)-β supports immune tolerance and regulates IGFBP expression, thereby modulating IGF availability [23,134]. TGF-β blocks the activation of quiescent naïve T cells into pro-inflammatory T cells, simultaneously promoting the differentiation and stability of Tregs [36,158,159]. TGF-β also stimulates IGFBP-3 secretion, with protease activity cleaving these binding proteins to locally regulate IGF-I availability [16,88,160]. Furthermore, in CRC stroma, TGF-β acts in synergy with autocrine IGF-II signaling to drive myofibroblast differentiation and promote a pro-invasive microenvironment [47]. During inflammation and tumor progression, TGF-β acts together with IL-10, often secreted by IGFBP-2-polarized M2 macrophages in pancreatic cancer, to inhibit pro-inflammatory cytokine production and enhance immunosuppressive pathways, thereby limiting effector T cell activation, as demonstrated for CRC and pancreatic cancer [100,161]. This illustrates how anti-inflammatory pathways interact with the regulation of the IGF/IGFBP system to facilitate tumor immune evasion [90,130].

##### Effects of Acute vs. Chronic Inflammation

Acute inflammation is commonly associated with a temporary reduction in circulating IGF-I levels, as documented in cystic fibrosis and childhood chronic inflammatory diseases [131,162]. This change represents an adaptive metabolic response to tissue injury and the initiation of immune activation. In contrast, in CRC, tumor-induced chronic inflammation results in the persistent exposure of the TME to inflammatory cytokines [29,163,164]. This chronic inflammatory state remodels IGF signaling within the TME by promoting IGFBP remodeling, enhanced receptor signaling, and activation of compensatory growth factor pathways despite the suppression of IGF-I levels [16,50,84,165].

### 2.2. Immune Cell-Mediated Remodeling of IGF Bioavailability

#### 2.2.1. Tumor-Associated Macrophages (TAMs)

The TME exhibits dynamic macrophage plasticity, with transitions between antitumorigenic M1 and pro-tumorigenic M2 phenotypes functionally linked to the IGF system [157,166]. As described in Section 2.1.2, the cytokine-mediated modulation of IGFBP-2 and -4 secretion in colon cancer epithelial cells reshapes local IGF-I availability at the tumor site [108]. In parallel, EGFR signaling in tumor cells also promotes secretion of IGF-I into the local microenvironment [81]. This cytokine- and growth factor-mediated crosstalk represents a key mechanism through which innate immune activation and tumor-derived signals reshape IGF bioavailability, with downstream consequences for macrophage and dendritic cell polarization, as discussed in Section 3.1.4 and Section 3.1.5.

#### 2.2.2. T Lymphocytes

T lymphocytes both respond to and regulate the IGF system through mechanisms distinct from TAM-mediated cytokine secretion and IGFBP modulation. IGFBPs directly influence T cell infiltration and function. IGFBP-3 expression correlates negatively with CD8+ T cell infiltration in murine breast cancer tumor models, suggesting that IGFBP-3 suppression permits greater T cell accumulation [88]. Although IGFBP-3 is the most abundant circulating IGF binding protein and primarily functions to sequester IGFs, its specific mechanisms of T cell inhibition remain incompletely understood [88]. In CRC, metastasis-associated fibroblast (MAF)-derived IGFBP-2 suppresses both proliferation and CD25 expression in CD4+ and CD8+ T cells through mechanisms that appear to be independent of IGF sequestration [101]. This positions MAFs as critical stromal regulators of both IGF bioavailability and T cell suppression in metastatic CRC [101].

#### 2.2.3. MDSCs

MDSC recruitment and expansion in CRC depend on inflammatory cytokines, including IL-6 and signaling pathways, which activate STAT3 signaling, which intersects functionally with IGF-1R pathways [129,167]. Additionally, studies on murine tumor models of esophageal cancer have demonstrated that MDSCs expressing high CD38 levels have enhanced immunosuppressive capacities, and that this expression is induced by tumor-derived factors, including IL-6 and IGFBP-3 [104]. The direct effects of IGF signaling on MDSC survival, expansion, and immunosuppressive function are discussed in Section 3.1.6.

#### 2.2.4. Neutrophils and Granulocytes

Neutrophils and granulocytes regulate IGF bioavailability primarily through proteolytic mechanisms. Together with MMPs, neutrophil-derived serine proteases, including azurocidin and cathepsin G, degrade IGFBPs-1, -2, and -4, reducing their affinity for IGFs and releasing these bioactive growth factors into the TME, a process characterized in breast cancer and general biochemical models [88,95]. In addition to proteolytic mechanisms, reactive oxygen species (ROS) associated with inflammation induce oxidative modifications of IGFBP-2 and its binding partner α2-macroglobulin (α2-M) in CRC patients [98,168]. This oxidation reduces the binding affinity of the IGFBP-2/α2-M complex, favoring the formation of more readily transportable IGF/IGFBP-2 binary complexes that increase local IGF bioavailability [98,168]. Tumor-associated neutrophils establish a pro-tumorigenic environment through release of both proteases and inflammatory mediators [169,170], while IGF-I reciprocally supports neutrophil survival by blocking Fas-mediated apoptosis via the PI3K pathway in human granulocytes under physiological and inflammatory conditions [34,151].

### 2.3. Metabolic Competition and IGF Axis Modulation

#### 2.3.1. Nutrient Competition in TME

Cancer cells in CRC utilize aerobic glycolysis to generate biosynthetic intermediates, depleting glucose from the microenvironment and impairing T cell and natural killer (NK) cell effector functions [84,171,172]. This metabolic shift provides biosynthetic intermediates essential for rapid cell division rather than maximizes ATP production [84]. High glucose consumption by tumor cells depletes this nutrient from the microenvironment, establishing conditions that promote immune evasion [171,172].

IGF-1R activation enhances glucose transporter 1 (GLUT1) expression, which is commonly upregulated in aggressive CRC and correlates with poor clinical outcomes [84,173]. While tumor cells increase glucose uptake through GLUT1 upregulation, effector T cells are forced into metabolic insufficiency that impairs their cytotoxic function, a mechanism demonstrated in sarcoma models [174,175]. In contrast, Treg cells utilize alternative metabolic pathways, including fatty acid oxidation, that allow them to maintain a suppressive capacity in this nutrient-depleted environment, as shown in CRC, melanoma and other tumor models [176,177]. The metabolic alterations driven by the insulin/IGF-I axis thus create conditions that disadvantage antitumor immunity while favoring immunosuppressive populations.

Amino acid metabolism also contributes to metabolic competition within the TME. Tumor cells deplete glutamine and methionine, impairing dendritic cell maturation and T cell responses [171]. Amino acid availability additionally regulates IGF signaling at a systemic level, as hepatic amino acid uptake stabilizes IGF-I mRNA expression [84]. In CRC with metastases to the ovaries, malignant cells depend on glutamine supplied by retinol-binding protein-1-positive (RBP1+) myofibroblasts, with inhibition of the glutamine transporter ASCT2 blocking fibroblast-mediated tumor cell proliferation [178]. Glutamine competition similarly affects dendritic cell function, as glutamine uptake via SLC38A2 is essential for type 1 conventional dendritic cell antigen presentation capacity, as demonstrated in melanoma and colon cancer [179].

Nutrient deprivation triggers hyperphosphorylation of IGFBP-1 via protein kinase Cα (PKCα), increasing IGFBP-1 affinity for IGF-I and reducing free IGF-I bioavailability, a mechanism characterized in hepatocellular carcinoma models [88,96]. This post-translational modification represents an additional mechanism through which metabolic stress limits growth factor availability in the nutrient-depleted TME. In contrast, protein restriction reduces circulating IGF-I levels and can inhibit carcinogenesis in colon cancer, as well as in breast, prostate, and bladder models [84,163,166]. The systemic reduction in IGF-I associated with amino acid restriction may thus have dual effects, limiting tumor growth but potentially impairing dendritic cell-mediated T cell priming.

#### 2.3.2. Hypoxia and Hypoxia-Inducible Factor 1-Alpha (HIF-1α)

Inadequate oxygen delivery to rapidly growing tumor tissue generates hypoxic regions within the CRC microenvironment that interact extensively with IGF signaling [29]. Hypoxia occurs in approximately 50–60% of solid tumors, driving metabolic reprogramming, chemoresistance, and metastatic progression through HIF-1α [180]. In CRC cells, IGF-I stimulates HIF-1α protein synthesis through the PI3K/AKT and MAPK signaling pathways, promoting angiogenesis and metabolic adaptation [84,181].

IGFBP-2 participates in a feedback loop with HIF-1α that may sustain tumor proliferation under hypoxic stress. IGFBP-2 can stimulate HIF-1α expression, and HIF-1α reciprocally upregulates IGFBP-2 in low-oxygen conditions in various cancer types [102,182]. In CRC, elevated circulating IGFBP-2 levels may partly reflect this hypoxia-driven upregulation [57,99]. Evidence further suggests that IGF-I/IGF-1R activation under hypoxia supports cancer stem cell maintenance and protects against oxidative stress in the CRC TME and other cancers [183,184]. In contrast, hypoxia induces IGFBP-6 expression in endothelial cells, which plays a negative role in tumor angiogenesis, as demonstrated in rhabdomyosarcoma models and other experimental models [111,112], demonstrating that hypoxia-induced IGFBP responses are heterogeneous and context-dependent.

#### 2.3.3. Metabolic Byproducts

**Figure 2:**
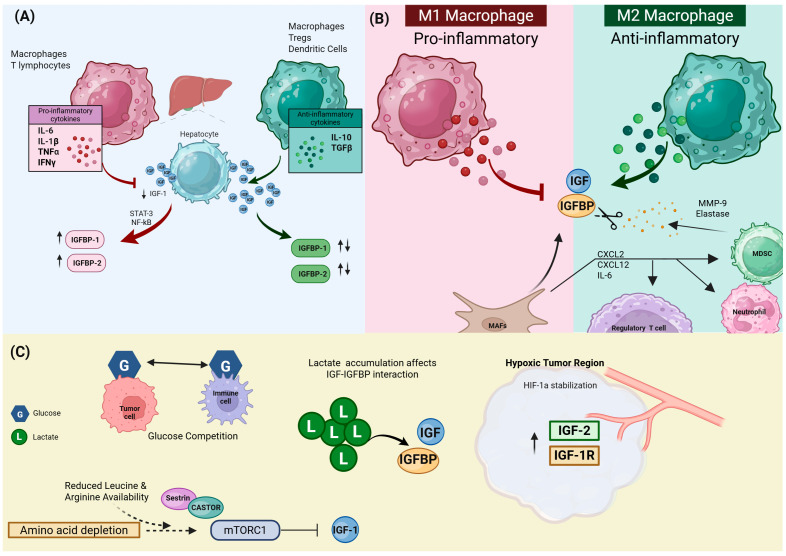
The immune regulation of the IGF/IGFBP axis in the colorectal cancer microenvironment. This figure illustrates multiple layers of the immune-mediated regulation of IGF bioavailability in the CRC tumor microenvironment through three interconnected regulatory mechanisms. (**A**) The cytokine-mediated modulation of systemic and local IGF production: Pro-inflammatory cytokines (IL-6, TNF-α, IFN-γ, IL-1β) suppress IGF-I production and modulate IGFBP expression through STAT3 and NF-κB signaling. Anti-inflammatory mediators (IL-10, TGF-β) exert context-dependent bidirectional effects on IGFBP activity. (**B**) Immune cell-dependent regulation of tumoral IGF: M1 macrophages paradoxically increase local IGF bioavailability by reducing IGFBP secretion in CRC epithelial cells, while M2 macrophages, neutrophils, and MDSCs enhance IGF bioavailability through IGFBP cleavage and direct IGF-I/IGF-II production. MAFs represent a critical source of immunosuppressive IGFBP-2. (**C**) Metabolic competition effects within the tumor TME: glucose competition between tumor cells and immune cells, lactate-mediated pH effects on IGF-IGFBP binding, hypoxia-driven HIF-1α/IGF-II upregulation, and amino acid depletion suppressing IGF-I production via mTOR inhibition. Created in BioRender. Zenón, C. (2026) https://BioRender.com/et822j, accessed on 20 March 2026.

Lactate accumulation and extracellular acidification in the CRC microenvironment reshape both immune function and IGF system activity [84,172]. Acidosis polarizes TAMs toward immunosuppressive phenotypes, impairs cytotoxic T cell and NK cell function, and suppresses IFN-γ production, mechanisms documented in lung and ovarian cancer [172,185,186]. Acidification also alters IGFBP function and distribution within the TME. IGF-II/IGFBP-2 complexes bind with greater affinity to ECM glycosaminoglycans at low pH, potentially concentrating IGFBP-2 in the tumor ECM, including CRC [103,187]. These crosstalk mechanisms are summarized in Figure 2.

## 3. IGF Axis Modulation of Immune Responses

### 3.1. IGF Signaling Effects on Immune Cell Function

In Section 2, we discuss the roles of tumor immune cells and inflammatory mediators in governing the availability, stability, and action of IGF network components in the context of CRC. Here, we cover the reciprocal impact of IGF signaling on tumor immune cell differentiation, metabolism, survival, and effector function.

#### 3.1.1. IGF Signaling in T Lymphocyte Function and Exhaustion

##### Survival, Proliferation, and Differentiation of CD4+ T Helper Cells

Naïve CD4+ cells express higher levels of IGF-1R compared to memory T cells, suggesting a specific role for IGF-I in early T cell development and differentiation [34,89]. IGF-1R expression is upregulated following T cell receptor (TCR) stimulation on CD4+ T cells, linking IGF-I signaling with enhanced cellular metabolism, proliferation, and survival following activation in both physiological and TMEs [34,89,188]. Alongside cytokine-mediated TCR activation, IGF-I-driven oxidative phosphorylation is critical to meet rising energy demands during CD4+ T cell lineage commitment. Under inflammatory conditions, this crosstalk between the TCR and IGF-1R signaling networks favors the differentiation of Th17 cells in non-tumor models [36]. IGF-I drives the pathogenic fate of Th17 cells by upregulating hexokinase II, further enhancing glycolysis and oxidative phosphorylation while reducing the mitochondrial membrane potential to mitigate the production of ROS [36,188]. In addition to its influence on Th17 differentiation, sustained IGF-I signaling enhances Treg stability, enabling these cells to modulate antigen-presenting cell function. Altogether, these findings clearly indicate that the metabolic reprogramming mediated by IGF-I actively contributes to the determination of the cell fate in CD4+ T cells across inflammatory and autoimmune environments [35,36,89,188]. Specifically in CRC, IGF/IGF-1R signaling restricts CD4+ and CD8+ T cell responses through extrinsic pathways; targeting this axis in IGF-II-high CRC models significantly restores the infiltration and antitumor function of tumor-infiltrating CD4+ and CD8+ T cells by suppressing MDSCs [82].

##### Activation and Exhaustion of CD8+ Cytotoxic T Cells

Several studies have shown that IGF signaling supports T cell maturation and proliferation. Specifically, IGF-I has been shown to promote the phenotypic conversion of naïve CD8+ T cells to a mature/memory phenotype in vitro using human cord blood and autoimmune models [151,189]. In activated T cells, IGF-1R-mediated pathways can provide a metabolic effect that supports aerobic glycolysis and nutrient uptake in CD4+ models of autoimmunity, whereas in CRC, this IGF-driven aerobic glycolysis predominantly occurs in tumor cells, metabolically restricting the T cells [36,84,188]. However, chronic IGF-1R activation in the TME is strongly associated with immune evasion and the suppression of cytotoxic CD8+ T cell function in CRC and breast cancers [82,90]. IGF-1R signaling in lung and breast cancers has been linked to the sustained expression of inhibitory receptors, including programmed death-1 (PD-1), on tumor-infiltrating lymphocytes. Additionally, IGF-1R inhibition reduces the expression of exhaustion markers, including PD-1 and glucocorticoid-induced TNFR-related protein (GITR), on tumor-infiltrating CD4+ and CD8+ T cells in lung cancer [34,87]. In summary, IGF signaling may boost early immune responses against tumors, but persistent pathway activation is associated with declining intratumoral immune function over time.

##### Memory T Cell Formation

Generation of memory T cells requires a metabolic change from glycolytic towards oxidative phosphorylation and oxidation of fatty acids [190,191]. In the CRC TME, this metabolic transition is frequently disrupted. High levels of stromal- and tumor-derived IGF-II in CRC and other malignancies result in the chronic activation of the IGF-1R pathway in infiltrating T cells [24,82]. This sustained signaling maintains high PI3K/AKT/mTOR signaling activity, which forces T cells to retain a glycolytic phenotype and inhibits the lipid metabolism necessary for memory differentiation [192,193]. Instead of developing into long-lived cells, CD8+ T cells in IGF-II-high CRC tumors are driven toward terminal exhaustion or senescence, characterized by mitochondrial dysfunction and poor antitumor efficacy [82,193]. Targeting this pathway to dampen mTOR activity or promote fatty acid oxidation could restore the metabolic flexibility required for memory cell formation in CRC. Together, these findings suggest the importance of the level of IGF signaling, and not necessarily its presence, in influencing the quality of memory T cell production.

#### 3.1.2. Regulatory T Cells

Tregs are pivotal regulators of the immune system, playing a dual role in maintaining peripheral tolerance and preventing autoimmune diseases. However, in the context of cancer, they participate in tumor development and progression favoring the TME and suppressing the antitumoral response [194]. Beyond its classic role as a growth factor, IGF I signaling can enhance Treg proliferation by regulating the PI3K/AKT/mTOR pathway in autoimmune disease models [35]. Mechanistically, PI3K/AKT/mTOR activation upon IGF-I binding promotes proliferation, yet this same pathway can suppress de novo FOXP3 induction in naïve CD4+ T cells [195,196]. Nevertheless, in committed Tregs, IGF-I synergizes with IL-2 to stabilize the Treg phenotype and support their expansion within the inflammatory milieu in autoimmune conditions [34,89].

Both IGF ligands and IGFBPs contribute to Treg differentiation and tumor immune tolerance. Studies on autoimmune models and malignancies such as hepatocellular carcinoma indicate that IGF-I stimulates Treg proliferation and enhances immunosuppressive capacity [34,35,166], while IGFBP-2 drives Treg differentiation and immunosuppression through STAT3 signaling in pancreatic cancer [100,167]. In metastatic CRC, fibroblast-derived IGFBP-2 has been shown to suppress T cell proliferation [101]. This convergence of the IGF-I/PI3K/AKT and IGFBP-2/STAT3 pathways positions the IGF network as a regulatory hub linking inflammatory signaling to immune tolerance in CRC pathogenesis [24,29,36].

#### 3.1.3. NK Cells

IGF-I supports the development and maturation of NK cells by promoting the survival of progenitors and inducing cytolytic activity during acute activation [145,197,198]. However, the role of IGF-I signaling in tumor-associated NK cells is yet to be elucidated. In CRC and non-CRC tumors, high levels of IGF ligands contribute to an immunosuppressive microenvironment that indirectly inhibits cytotoxic effector cells [24,34]. IGF-I and -II promote the recruitment and activation of immunosuppressive myeloid-derived populations, which are inhibitors of NK cell function [34,82]. In IGF-II-high CRC, MDSCs have been identified as the primary cell type expressing high levels of IGF-1R. The activation of this receptor enhances their recruitment and suppressive activity against T cells in CRC [82] and NK cells in other cancer types [199].

In certain TMEs, high IGF-I may indirectly inhibit NK cell function by supporting the recruitment of immunosuppressive stromal and myeloid cells. While physiological IGF-I generally promotes NK cell development and cytotoxicity, in the TME, high IGF-I levels can indirectly impair NK function by fostering an immunosuppressive landscape through expansion of M2 macrophages, Tregs, and MDSCs that secrete factors such as TGF-β known to downregulate NK activating receptors [24,34,127,199]. Evidence from other gastrointestinal malignancies supports this mechanism; in hepatocellular carcinoma, miR615-5p directly represses IGF-1R expression in NK cells, diminishing their cytotoxic capacity and facilitating tumor immune escape [200]. Whether a similar miRNA-mediated suppression of NK cells through IGF-1R targeting operates in the CRC microenvironment has not been investigated, but this represents a plausible mechanism given the established role of IGF-1R signaling in shaping innate immune responses within colorectal tumors.

#### 3.1.4. Dendritic Cells

IGF-I has been shown to suppress dendritic cell maturation and the antigen-presenting capacity in ovarian cancer models, maintaining them in a phenotype characterized by the reduced expression of co-stimulatory molecules [34,201]. While IGF-II can induce dendritic cell maturation, it often promotes a tolerogenic phenotype associated with IL-10 secretion that dampens T cell priming efficiency [23]. These mechanisms of signaling can cause an immune-suppressive dendritic cell phenotype characterized by an increase in IL-10 production and reduced inflammatory cytokine secretion, which, in turn, favors Tregs and suppresses effector T cells [147,165]. Particularly within MSS CRC tumors, dendritic cells are frequently skewed toward this tolerogenic state by TME factors including TGF-β, vascular endothelial growth factor (VEGF), and IL-10 [127,202]. IGF-II signaling further compounds this dysfunction by upregulating PD-L1 and stromal cell-derived factor 1 (CXCL12) in the stromal compartment of breast cancer and CRC, creating an immune-excluded environment that disrupts the dendritic cell–T cell crosstalk necessary for effective antitumor immunity [90]. Additionally, IGF signaling contributes to immune evasion in prostate cancer through downregulation of antigen-processing machinery, including transporters associated with antigen processing (TAP1/2) and β2-microglobulin [93] In CRC, tumor-associated dendritic cells exhibit deficient antigen presentation and elevated IL-10 secretion, and CXCL1-overexpressing dendritic cells derived from CRC patients actively promote cell motility, epithelial–mesenchymal transition, and cancer stemness [155].

#### 3.1.5. Macrophages

The accumulation of local IGF-I generated through immune cell-mediated IGFBP remodeling (Section 2.2.1) directly shapes the macrophage phenotype within the CRC TME. IGF-I/IGF-1R signaling promotes macrophage polarization from M1 toward M2 through activation of AKT and downstream metabolic pathways, favoring an anti-inflammatory, pro-tumorigenic phenotype characterized by elevated IL-10 production and increased arginase activity in vitro and in various cancers, including colon cancer [34,81,83]. In mouse colitis-associated tumorigenesis models, treatment with the EGFR inhibitor cetuximab decreases F4/80+/CD206+ M2 macrophage populations and downregulates the M2 phenotype-associated markers Arg1, IL-10, and IL-4 [81].

Macrophage polarization within the TME is ligand- and receptor-dependent. While IGF-I/IGF-1R signaling drives polarization toward pro-tumorigenic phenotypes (M2) [34], low-dose IGF-II/IGF-2R signaling can reprogram macrophages toward an anti-inflammatory, immunosuppressive, oxidative phosphorylation-dependent phenotype via GSK3 signaling [23,91,92,203]. In addition to IGF-1R signaling, IGF-II can modulate macrophage polarization and tissue remodeling in non-neoplastic inflammatory models [91,92], though its potential action through the IR-A in macrophages warrants further investigation in cancer [23]. Within breast cancer and other solid tumors, TAMs are known to be major sources of IGF ligands that contribute to angiogenesis, ECM remodeling, and immune evasion [22]. Macrophages also produce and respond to IGF ligands, establishing autocrine and paracrine feedback loops that sustain macrophage survival and tumor-promoting activities [86]. These IGF-I-driven TAMs secrete growth factors, including IGF-I and -II, and cytokines that further amplify M2 polarization [204].

IGFBP-2 coordinates an immunosuppressive network across multiple cancers. Potentiating nuclear EGFR/STAT3 signaling, IGFBP-2 promotes the upregulation of PD-L1 expression in several cancer types [100,103]; it is also a downstream target NF-κB pathway that sustains inflammation and tumor progression in CRC [205]. Through STAT3 signaling activation, IGFBP-2 induces M2 macrophage polarization while increasing IL-10 expression and secretion in pancreatic cancer models [100]. High IGFBP-2 levels correlate with M2-polarized TAM accumulation and a poor prognosis in pancreatic cancer [100], with studies in glioblastoma demonstrating that IGFBP-2 inhibition increases CD8+ T cell infiltration while decreasing M2 macrophages [206]. The mechanism involves the STAT3-mediated upregulation of IL-10, which inhibits T cell antitumor immunity and impairs T cell infiltration [100]. Other IGFBPs also show context-dependent roles in macrophage biology; in glioma, IGFBP-5 expression positively correlates with M2 macrophage infiltration and programmed death-ligand 1 (PD-L1) expression, suggesting an additional binding protein that contributes to immunosuppressive macrophage phenotypes [110]. M2-polarized TAMs interact with other immunosuppressive populations, including MDSCs and Treg cells, integrating multiple layers of immune regulation that collectively reduce cytotoxic lymphocyte activity, as demonstrated in non-small-cell lung cancer [207]. Through these interdependent networks, IGF signaling amplifies the immunosuppressive landscape, enhancing tumor cell survival, angiogenesis, and matrix remodeling while limiting effective antitumor immunity [24].

#### 3.1.6. IGF Signaling in MDSCs

MDSCs express high levels of IGF-1R, and IGF signaling directly supports MDSC survival and tumor-promoting functions through IGF-1R in CRC [82], with IGF pathway inhibition inducing apoptosis in MDSC populations. IGF-I can promote the expansion and migration of these cells by engaging IGF-1R on progenitor and circulating myeloid populations, inducing survival and chemotaxis in CRC and other solid tumors [82,199]. IGF-I supports MDSC activation by supporting the PI3K-AKT and MAPK signaling pathways across various cellular contexts, which upregulate immunosuppressive intermediaries such as arginase-1, inducible nitric oxide synthase (iNOS), and ROS [18,26,208]. These molecules enhance the MDSC capacity to inhibit T cell proliferation and effector function, reduce NK cell activity, and promote Treg expansion. This IGF-induced MDSC immunosuppressive function helps tumors evade immunity, support angiogenesis, and remodel tissue, leading to an immune-suppressive microenvironment and driving tumor progression across diverse malignancies, including CRC [209,210].

### 3.2. IGF Axis and Immune Checkpoint Expression

#### 3.2.1. PD-1/PD-L1

PD-L1, also known as B7-H1 or CD274, is a transmembrane protein and the principal ligand molecule of the inhibitory receptor programmed death-1 (PD-1/CD279) [211]. PD-L1 can be expressed constitutively at lower levels on resting antigen-presenting cells and certain epithelial cells and at higher levels on many tumors, including CRC [212,213,214,215]. PD L1 expression is mainly controlled by inflammatory cytokines such as IFN γ and oncogenic signals (EGFR, MAPK and PI3K/AKT) and is further regulated by hypoxia mechanisms and transcription factors, including STAT3 and HIF 1 [211,215,216]. In various cancers, activated T cells express higher levels of PD-1, prompting PD-L1 expression in nearby tissues by releasing IFN γ and TNF α [217,218].

IGF 1R and IR-A share ligands and downstream effectors, creating a receptor network that combines nutrient and growth signals to promote survival and immune modulation [22,23]. PI3K/AKT activation enhances PD L1 transcription and protein stability in multiple cancers, and this effect can occur at both the transcriptional and post-transcriptional levels, often independently of mTOR in colon cancer [41,219].

Transcription factors such as STAT3 and HIF-1 bind directly to PD-L1 promoter elements and are themselves regulated by PI3K/AKT and ERK signaling. These pathways modulate the transcriptional activity of STAT3 and HIF1, facilitating the recruitment of these transcription factors to the PD-L1 promoter in various cancer types [146,216]. Furthermore, NF-κB, which is activated downstream of Toll-like receptors, cytokine receptors, and PI3K/AKT, becomes a major mediator of IFN γ-induced PD L1 and can be blocked pharmacologically to reduce PD L1 expression [41]. Both pathways are interconnected, as AKT can modulate NF-κB, and activator protein 1 (AP-1) cooperates with STAT3 at the PD L1 promoter, forming a regulatory mechanism through which IGF-1R and IR-A are translated into durable PD-L1 upregulation in melanoma, prostate cancer, and pancreatic cancer [23,24,93,220]. Beyond these transcriptional mechanisms, post-transcriptional regulation through ncRNA provides an additional layer of PD-L1 control relevant to IGF signaling. In non-CRC models, circRNAs, including circRHBDD1 and circATAD2, stabilize PD-L1 mRNA by binding to IGF2BP proteins [58], while in CRC, hsa_circ_0020397 acts as an miRNA sponge to upregulate PD-L1 [221], and the miRNA-mediated regulation of PD-L1 has also been described [222], potentially linking IGF axis post-transcriptional regulation to immune checkpoint evasion.

In chronic infections and cancer, PD 1 upregulation and high PD L1 expression drive a stable state of T cell dysfunction, or “exhaustion.” This dysfunction is now well recognized as a hallmark of ineffective antitumor immunity, often associated with an immunologically “cold” tumor phenotype characterized by poor T cell infiltration [121,161]. Mechanistically, sustained PI3K/AKT/MAPK signaling and inflammatory cytokines trigger high PD L1 production in tumor tissues, maintaining continuous PD 1 engagement, while regulatory populations and immunosuppressive cytokines further enforce exhaustion [215]. In melanoma and lung cancer, responses to PD-1 therapy require the presence of PD-1+ CD8+ T cells and PD L1-expressing cells in the TME, consistent with the idea that reversing PD-1 exhaustion at the tumor site is a key mechanism of action [215,223].

#### 3.2.2. Other Immune Checkpoints

The cytotoxic T lymphocyte associated antigen 4 (CTLA-4) is a transmembrane receptor of the CD28 family that binds to the same ligands as CD80 (B7-1) and CD86 (B7-2) on antigen-presenting cells but with higher affinity than CD28 [224,225]. Unlike PD-1/PD-L1, the CTLA-4 immune checkpoint functions primarily during T cell activation in lymphoid organs, favoring the immunosuppressive role of Tregs cells [225,226]. IGF-I promotes Treg proliferation and expansion, synergizing with IL-2 through IGF-1R/PI3K/AKT in non-tumor autoimmune models, expanding this suppressive population that constitutively expresses CTLA-4 [34,35,89]. This is relevant in the TME, where the dysregulated proteolysis of IGFBPs enhances the local IGF bioavailability [24,80]. Furthermore, targeting CTLA-4 remains one of the most effective therapy approaches against cancer when using a combination of anti-CTLA4 (ipilimumab) and anti-PD-1 (nivolumab) in melanoma [227,228], but this efficacy is largely restricted for deficient MMR and MSI-H CRC [125,127,228]. In summary, the IGF axis acts as a central modulator of CTLA 4 function, promoting an immunosuppressive microenvironment by enhancing Tregs via the IGF-1R/AKT/ERK signaling pathway and upregulating immune checkpoints like PD-1 [34,90].

### 3.3. Impact on TME Architecture

IGF signaling plays a role in shaping the structural and functional organization within the TME by coordinating immune cell recruitment, vascular remodeling, and ECM dynamics, with supporting evidence from bone sarcoma and other solid tumors [34,137,160]. When IGF pathways are activated in tumor and stromal cells, they alter chemokine expression patterns and, consequently, influence the composition and location of infiltrating immune cells, as demonstrated for CRC and non-CRC models [90,229]. IGF signaling promotes recruitment of immunosuppressive cells, such as MDSCs, while limiting infiltration of CD8+ T cells in the TME of CRC, breast cancer, and other solid tumors [34,82,90]. In this way, the IGF pathway contributes to immune exclusion and an immunologically “cold” tumor phenotype, characterized by reduced or functionally impaired immune cell infiltration and poor responsiveness to immunotherapies.

Additionally, in both CRC and non-CRC models, IGF signaling intersects closely with angiogenesis and vascular remodeling, influencing accessibility of the TME to the immune system [180,230]. Through induction of VEGF and related pro-angiogenic mediators, IGF pathways promote the formation of new blood vessels that support tumor growth and metabolic needs across a variety of malignancies, including CRC, as well as thyroid and pancreatic cancers [119,231]. However, as observed in CRC and other cancers, these blood vessels are generally structurally abnormal, poorly organized, and dysfunctional. The resulting vascular dysfunction can contribute to hypoxia and endothelial barriers that affect immune cell infiltration [128,232]. Endothelial VEGF signaling can suppress adhesion molecule expression and promote immune inhibitory signals that can limit T cell trafficking in the presence of tumor-specific immunity across these diverse tumor types [121,233].

**Figure 3:**
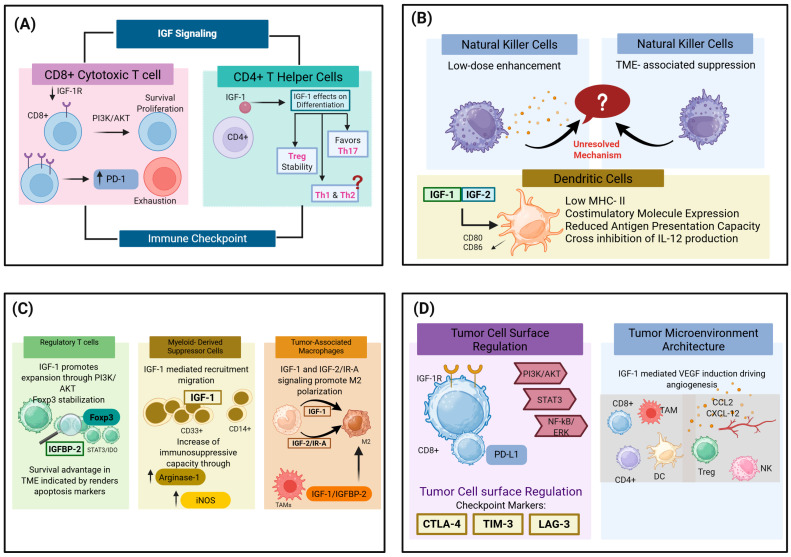
IGF/IGFBP axis modulation of immune cell function and checkpoint expression in colorectal cancer. This figure illustrates how IGF signaling affects immune cell populations and checkpoint regulation in CRC. (**A**) Dual effects of IGF-I on T lymphocytes: low-level signaling supports CD8+ survival and proliferation, while sustained IGF-1R activation drives PD-1 upregulation and exhaustion; in CD4+ T helper cells, IGF-1 favors Th17 differentiation and Treg stability. (**B**) Innate immune cells: physiological IGF-1 signaling enhances NK cell cytotoxicity, whereas TME-associated levels indirectly suppress NK function; IGF-I/II maintain dendritic cells in tolerogenic state with reduced antigen presentation capacity. Question mark symbol indicates an unresolved mechanism. (**C**) Immunosuppressive cell populations: Treg proliferation via PI3K/AKT/mTOR signaling with IGFBP-2/STAT3-driven differentiation, MDSCs recruitment with enhanced arginase-1/iNOS activity, and M2 TAM polarization through reciprocal IGF-I/IGFBP-2 amplification loops. (**D**) Checkpoint regulation and TME architecture: convergence of IGF-1R signaling (PI3K/AKT, STAT3, NF-κB, ERK) on PD-L1 upregulation, CTLA-4 mediated immunosuppression through IGF-driven Treg expansion, and TME architectural remodeling, including VEGF-driven angiogenesis, altered chemokine gradients and CAF-mediated T cell exclusion. Created in BioRender. Zenón, C. (2026) https://BioRender.com/gs84yfb, accessed on 20 March 2026.

IGF signaling regulates the ECM and stromal microenvironment by activating CAFs, which remodel the basement membrane and physically generate microtracks in the ECM through force-mediated interactions, facilitating cancer cell migration and invasion. Additionally, CAFs alter ECM stiffness and cell–matrix interactions promoting angiogenesis and enhancing tumor progression. Chemokines secreted by CAFs contribute to the enhancement of the immune exclusion of effector lymphocytes through the retention of the lymphocytes in the periphery and the activation of immunosuppressive myeloid cells in breast, pancreatic, CRC, and other cancer models [90,229,233,234,235]. By restructuring the ECM, influencing blood vessels and immune cell positioning, IGF signaling helps tumors evade the immune system, grow, and resist therapy, making this pathway a central player in tumor architecture and an attractive target for treatments aimed at the TME [24,34,90,229,236]. These interactions are illustrated in Figure 3.

## 4. Bidirectional Crosstalk and Context Dependency

### 4.1. Feedback Loops and Regulatory Circuits

The individual mechanisms through which immune signals regulate IGF bioavailability and IGF signaling modulates immune function do not operate independently but converge into feedback circuits whose net effect determines tumor immune evasion and therapeutic resistance in colorectal, breast, and ovarian cancers [90,218,237]. IGF signaling mediates immunosuppressive myeloid programs, suppresses antigen presentation, and promotes checkpoint ligand expression, while the resulting immune suppression allows tumor and stromal cells to maintain local IGF activity, establishing self-sustaining loops in CRC, pancreatic cancer, and other TMEs [24,82,90,93,100,143,229].

Taken together, these coupled feedback processes form a complex, nonlinear regulatory circuitry that can be sensitive to even small disturbances. Phenotypes such as immune exclusion and resistance to immunotherapy result from the integrated regulation of IGF signaling, cytokine networks, and immune checkpoint pathways across various solid tumors, including Ewing sarcoma, prostate cancer, and epithelial ovarian cancer [93,201,238]. These interconnected circuits help explain the failure of single-target therapies and argue for combination strategies that both block IGF signaling and restore antitumor immune responses.

### 4.2. Molecular Subtype-Dependent Interactions

#### 4.2.1. MSI-H vs. MSS Tumors

Despite the characteristic strong immunoreactivity profile of MSI-H CRC, IGF-induced immunomodulation in this cancer type has been relatively understudied [239]. In contrast, the IGF axis appears particularly critical in the MSS subtype and mesenchymal subtypes, where activation of IGF-1R signaling has been shown to silence innate immune sensors and promote immune evasion through mechanisms such as degradation of retinoic acid-inducible gene I [94]. Furthermore, high IGF-1R activity in CRC, and other types of cancer is associated with macrophage polarization toward pro-tumorigenic M2 phenotypes that suppress adaptive immunity [34,81]. Mechanistic studies on prostate cancer have shown that IGF-1R signaling can impair antigen presentation through the downregulation of components of the antigen-processing machinery (APM), including TAP1/2, endoplasmic reticulum aminopeptidase-1 (ERAP-1), and β2-microglobulin, thereby limiting T cell recognition even when neoantigens are present [93]. Although these specific APM alterations have been characterized in prostate models, they are relevant to CRC given that IGF-1R is similarly overexpressed and activates the same downstream PI3K/AKT signaling cascades implicated in immune evasion across breast, prostate, and other solid tumors [32,34,93]. This multi-layered immunosuppressive effect, encompassing both suppression of innate immune sensing and impaired adaptive immune recognition, positions the IGF system as a central mediator of immune evasion in the MSS subtype.

#### 4.2.2. Consensus Molecular Subtypes (CMSs)

Among the four CMS subtypes, CMS1 and 4 have the most extensively characterized IGF–immune interactions and are therefore the focus of this section. The remaining subtypes (CMS2, characterized by Wnt and MYC pathway activation with low immune infiltration, and CMS3, defined by epithelial differentiation and metabolic dysregulation) have not been extensively studied in this context [10,11,239], despite the known IGF pathway enrichment in CMS3 [50,240]. CMS1, also known as the MSI-immune subtype, comprises the majority of MSI-H tumors and is frequently associated with BRAF mutations [10,11]. This subtype is characterized by strong immune cell infiltration and activation of cytotoxic signaling pathways, which typically result in favorable outcomes for patients with early-stage disease [10,240]. Expected dynamics in CMS1 involve high levels of T cell-attracting chemokines and upregulation of immune checkpoints such as PD-L1 to counterbalance the vigorous immune microenvironment [239,240]. However, the role of IGF-induced immunomodulation remains underexplored.

The CMS4 mesenchymal subtype is characterized by a high degree of stromal infiltration, angiogenesis, activation of epithelial–mesenchymal transition, and a poor prognosis [11,240]. This subtype features prominent interplay between the stroma and the immune system, where CAFs secrete factors such as IGF-II and CXCL12 to induce T cell exclusion and foster immunosuppressive conditions across CRC, breast, and other tumors [90,239]. High levels of TGF-β signaling in the CMS4 microenvironment further support this interplay by activating CAFs and promoting recruitment of immunosuppressive cell populations [202,240].

#### 4.2.3. Tumor Stage Dependency

As CRC advances from the early to metastatic stages, the progression is marked by a steady increase in circulating IGFBP-2 levels [57,99], which coincides with significant shifts in immune composition [127,202]. While patients with early-stage disease often achieve successful surgical outcomes, those at advanced stages face considerably lower survival rates due to the increased tumor burden and systemic immune impairments [101,155]. Additionally, advanced-stage tumors often show decreased TILs compared to early-stage tumors, suggesting a process of progressive immune escape in CRC and non-CRC models [202,241]. Biological characteristics and immune dynamics also often differ substantially between primary tumors and metastatic sites. Fibroblasts from peritoneal metastases secrete higher levels of IGFBP-2 compared to primary tumor CAFs, with a greater capacity to suppress T cell function and polarize macrophages toward suppressive phenotypes. Metastatic sites tend to exhibit more pronounced immune-excluded landscapes compared to primary tumors in CRC, breast cancer, and other solid tumors [90,101].

### 4.3. Systemic vs. Local Regulation

#### 4.3.1. Systemic Effects

Systemic IGF-I, produced primarily by the liver, circulates bound to IGFBPs, with IGFBP-3 carrying approximately 85% of circulating IGFs [29]. Hepatic IGF-I production is stimulated by growth hormone and regulated by insulin levels, where insulin acts as a physiologic regulator of hepatic IGFBP synthesis [22,84]. Metabolic conditions alter the systemic IGF family through effects on both ligands and binding proteins. Chronic hyperinsulinemia associated with obesity and type 2 diabetes suppresses the hepatic production of IGFBP-1 and -2 [22,182]. IGFBP-1 expression is particularly insulin-sensitive, with regulation occurring through transcription factor FOXO1 [88]. This suppression of circulating IGFBPs increases the bioavailability of free IGF-I [84]. Insulin can also bind IGF-1R with low affinity to stimulate cell proliferation via PI3K/AKT/mTOR signaling across various malignancies, including CRC and breast cancer [22,23,84]. Chronic hyperglycemic conditions further activate this axis by enhancing GLUT1 glucose transporter expression and key glycolytic enzymes, including hexokinase 2 and lactate dehydrogenase A, thereby affecting glucose uptake and aerobic glycolysis in CRC cells [84].

Among pan-cancer metabolic reprogramming mechanisms, high-fat diets and obesity alter the fatty acid distribution in the TME of multiple solid tumors [171]. Specifically in CRC, this metabolic shift impairs the infiltration and function of antitumor T cells [242] while promoting Treg cell differentiation [124]. The interplay between a systemic metabolic state, circulating IGF/IGFBP levels, and immune cell priming represents an area requiring further investigation to understand how endocrine IGF signals shape antitumor immunity and T cell mitochondrial function across various malignancies, including CRC, prior to immune cell encounter with tumor antigens [34,93,188].

#### 4.3.2. Local Effects

The IGF system in tumors operates predominantly through local paracrine and autocrine mechanisms rather than through the tightly regulated endocrine circuit characteristic of normal physiology [29]. This local dysregulation creates high concentrations of IGF ligands and altered IGFBP profiles that directly shape immune cell behavior through proximity-dependent mechanisms distinct from systemic endocrine effects.

Tumor cells frequently produce high concentrations of IGF-II through epigenetic alterations such as loss of imprinting or reactivation of fetal promoters [63]. These alterations can generate high-molecular-weight IGF-II pro-peptides (Big IGF-II) with increased bioavailability and the capacity to activate signaling through IR-A in various solid tumors [23]. Tumor-produced IGFBPs like IGFBP-2 undergo intensive proteolytic degradation by proteases, including MMPs, ensuring that free IGFs remain available for receptor activation [29]. Locally produced IGF-II drives autocrine tumor cell proliferation while creating a paracrine environment that promotes immunosuppressive phenotypes in infiltrating myeloid populations.

Stromal cells represent critical local sources of IGF axis components that directly modulate immune function. In CRC and non-CRC models, CAFs increase secretion of IGF-II, which activates IGF-1R/yes-associated protein 1 (YAP1) signaling to drive progression, while promoting T cell exclusion via CXCL12 and PD-L1 upregulation [13,23,90]. MAFs produce substantially higher IGFBP-2 levels compared to primary tumor CAFs, contributing to local immunosuppression in metastatic sites of CRC and pancreatic cancer [100,101]. M2-polarized macrophages secrete IGF-I and IGF-II, enhancing cancer cell stemness and metastatic potential through PI3K/AKT/mTOR activation in thyroid, breast, and other cancers [24,157]. This creates bidirectional interactions where tumor and stromal-derived factors polarize macrophages toward an M2 phenotype, and these M2 macrophages then secrete IGFs that further support tumor progression while suppressing antitumor immunity. The spatial proximity of this paracrine crosstalk enables rapid, high-concentration signaling unattainable through systemic endocrine mechanisms [81].

In addition to the paracrine circuits described above, cellular senescence within the tumor stroma represents an emerging source of IGF system components that may further shape immune regulation in CRC. Senescent stromal cells acquire a senescence-associated secretory phenotype (SASP) characterized by secretion of pro-inflammatory cytokines, chemokines, proteases, and several IGF binding proteins, including IGFBP-2 through -6, now recognized as SASP components across various human cell types [243]. In CRC-relevant models, the transcriptomic profiling of primary human colon fibroblasts has established that IGFBP-2 is a universal component of the colon-specific SASP, secreted regardless of the senescence-inducing stressor [97]. Although SASP-mediated immunosuppression, including M2 macrophage polarization and regulatory T cell recruitment, has been documented in prostate, breast, and other solid tumors [244], the specific contribution of SASP-derived IGFBPs to immune evasion within the CRC microenvironment remains to be established.

#### 4.3.3. Gut–Systemic Interface: Considerations in CRC

**Figure 4:**
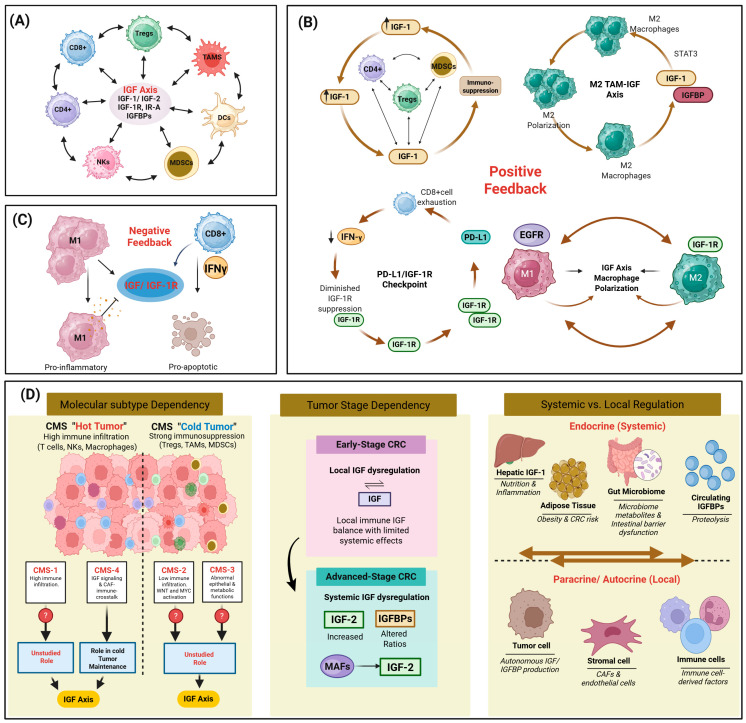
The bidirectional crosstalk and context-dependent regulation of IGF–immune interactions. This figure depicts the complex regulatory networks and context-dependent factors that govern IGF–immune interactions in CRC. (**A**) The core bidirectional network illustrating the reciprocal regulation between the IGF axis and immune cell populations. (**B**) Positive feedback loops: IGF-I-driven immunosuppression enables increased IGF-I production, M2 TAM-IGF/IGFBP-2/STAT3 amplification, and PD-L1/IGF-1R/IFN-γ circuit-perpetuating CD8+ exhaustion. (**C**) Negative regulatory mechanisms: IFN-γ-mediated suppression of IGF-1R expression and M1 macrophage cytokine secretion suppresses IGF bioavailability. (**D**) Context-dependency across three dimensions. Left: molecular subtype dependency distinguishes CMS1 (immune-infiltrated) and CMS4 (mesenchymal/stromal-rich) with characterized IGF–immune patterns, while CMS2 (canonical) and CMS3 (metabolic) lack direct evidence linking IGF activity to immune regulation. The question marks indicate understudied roles. Center: tumor stage dependency contrasting local IGF dysregulation in early-stage CRC with systemic dysregulation and MAF-driven immunosuppression in advanced/metastatic disease, where stromal fibroblasts, including MAFs contribute to altered IGF-II and IGFBP-2 levels in the metastatic microenvironment. Right: systemic versus local regulation, depicting the transition from hepatic IGF-I production to dominant paracrine/autocrine signaling by tumor cells, CAFs, and immune cells as disease progresses. Created in BioRender. Zenón, C. (2026) https://BioRender.com/8vjlj2u, accessed on 20 March 2026.

The relative contributions of systemic versus local IGF sources to immune regulation likely vary across tumor stages and molecular subtypes. In early-stage disease with a relatively intact tissue architecture, systemic IGF levels may play a more prominent role in shaping the immune microenvironment [84]. As tumors progress and develop a chaotic vascular architecture with impaired perfusion, local paracrine production by tumor and stromal cells becomes increasingly dominant. The rise of circulating IGFBP-2 during metastatic progression likely reflects both systemic responses to the tumor burden and the spillover of locally produced binding proteins into the circulation [57,99]. Given the anatomical position of CRC within the gastrointestinal tract, the interplay between the intestinal microbiota and host metabolism represents an additional consideration in this systemic–local balance. Microbial metabolites, particularly short-chain fatty acids such as butyrate, influence both systemic metabolic homeostasis and local immune function by modulating energy metabolism in colonocytes and shaping the differentiation of intestinal immune cells [245]. Recent single-cell transcriptomic analyses using colon cancer models have demonstrated that intact gut microbiota potentiates immune checkpoint inhibitor efficacy through macrophage lineage reprogramming, shifting tumor-associated macrophages from pro-tumoral Secreted Phosphoprotein 1 (SPP1) + phenotypes toward CD74+ antigen-presenting states, and by expanding effector-memory CD8+ T cells while restraining terminal exhaustion [246]. The antibiotic-mediated depletion of the gut microbiota significantly diminishes these immunotherapeutic responses in both murine models and clinical cohorts across various solid tumors, particularly melanoma, non-small-cell lung cancer, and other non-CRC malignancies [247,248]. While evidence indicates that intestinal epithelial IGF-1 is essential for maintaining the microbiome composition and preventing bacterial translocation [184], direct mechanistic studies establishing how microbiota-derived signals regulate tumor immunity specifically through the IGF network remain lacking, representing a significant knowledge gap that warrants the targeted investigation of CRC models. The core bidirectional regulatory networks discussed in Section 2, Section 3 and Section 4 are illustrated in Figure 4.

## 5. Biomarker Implications for Early Detection

The bidirectional and context-dependent nature of IGF–immune crosstalk carries direct implications for biomarker development. Because no single pathway component captures the full complexity of these regulatory circuits, effective detection strategies require the simultaneous assessment of circulating IGF axis components, immune cell signatures, and metabolic indicators. The measurability of the key nodes in these networks, including circulating IGFBP-2, immune cell infiltration patterns, and epigenetic modifications of IGF pathway genes, provides a foundation for multi-parametric approaches to early CRC detection.

### 5.1. IGF–Immune Signatures as Potential Biomarkers

#### 5.1.1. Systemic/Circulating Markers

Investigations into cancer risk frequently use the ratios of circulating IGFs to their binding proteins as functional indicators of system dysregulation. Higher circulating IGF-I concentrations coupled with lower IGFBP-3 levels are consistently associated with increased CRC risk across multiple population studies [163,249]. While these indicate risk, elevated serum IGF-II and IGFBP-2 may more accurately reflect tumor presence and burden while providing insight into cellular relationships governing growth factor bioavailability [57,99]. The chronic elevation of fasting and postprandial insulin and IGF levels further increases cancer risk by reducing binding proteins such as IGFBP-1 and -2, which enhances the bioavailability of free IGF-I capable of activating proliferative signaling cascades [84].

#### 5.1.2. Tissue-Based Markers

IGF-1R expression is frequently elevated in primary colorectal tumors, with protein levels increasing significantly as tumors progress through successive Dukes stages, advancing from early, localized lesions (Dukes A and B) to regional and distant metastatic disease (Dukes C and D) [45]. This receptor overexpression occurs along the entire sequence, from normal mucosa through adenomatous polyps to invasive cancer [45]. IGF-1R signaling within the TME suppresses antitumor immune responses while activating CAFs and recruiting immunosuppressive myeloid cells [129]. Other cancer studies show that IGF-I suppresses cancer cell recognition by immune effectors through the downregulation of APM, including transporters associated with antigen processing and β2-microglobulin, thereby compromising the MHC class I-mediated presentation of tumor antigens [93]. Tumors characterized by elevated endogenous IGF-I and IGFBP-5 demonstrate higher expression of PD-L1, indicating convergence between IGF network activation and immune checkpoint regulation [93].

IGFBP-2 has emerged as a particularly informative tissue biomarker given its differential secretion by fibroblasts within colorectal metastases and its functional role in shaping immunosuppressive microenvironments [101]. In CRC, elevated IGFBP-2 promotes immune evasion and its co-elevation in serum, with markers such as Chitinase-3-like Protein 1 (CHI3L1) supporting its role as an indicator of poor prognosis [250].

#### 5.1.3. Multi-Parametric Approaches

Integration of IGF axis components with molecular biomarkers offers substantial advantages over individual markers for CRC detection. A three-biomarker panel consisting of IGFBP-2, dickkopf-related protein 3 (DKK3) and pyruvate kinase M2 (PKM2) achieved seventy-three percent sensitivity at 95% specificity for discriminating CRC patients from healthy controls in an Australian cohort (*n* = 342) [251]. This demonstrates the value of capturing different aspects of tumor biology, including growth factor signaling, Wnt pathway antagonism, and metabolic reprogramming. This multi-biomarker approach also improved diagnostic accuracy and outperformed individual biomarkers in Hispanic CRC patients [252]. The combination of IGFBP-2 with carcinoembryonic antigen significantly increased the sensitivity for detecting metastases and recurrences compared to either marker individually [99]. Elevated plasma IGFBP-2 levels function as both diagnostic and prognostic biomarkers, demonstrating an independent association with increased mortality risk, which suggests utility for risk stratification beyond initial detection [57].

Machine learning algorithms have been adopted to develop diagnostic models that differentiate CRC patients from controls with high accuracy based on tumor RNA and molecular profiles [32]. Recent multi-omics analyses assisted by deep learning have identified macrophage-oriented signatures as potential therapeutic targets in CRC, demonstrating the capacity of these methods to synthesize immune and metabolic information [253,254]. The integration of artificial intelligence with multi-omics biomarker panels holds substantial potential for enhancing therapeutic targeting precision and improving patient stratification [32].

### 5.2. Methodological Considerations

The identification of reliable biomarkers for CRC encounters substantial obstacles related to variability in analytical methods and the absence of universal standards across diagnostic laboratories. Variability in antibody specificity for intact versus proteolyzed proteins such as IGFBP-2 introduces significant measurement discrepancies between assays due to differences in calibration curves, antibody standards, and immunoreactivity [98,255]. Additionally, the epigenetic silencing of IGFBP-3 through promoter methylation adds complexity to IGF axis biomarker development in CRC, requiring standardized DNA methylation assays to ensure reproducibility [29,256].

A fundamental challenge in the clinical application of growth factor signaling as biomarkers lies in the functional decoupling that can occur between systemic circulating levels and local activity within the TME. Circulating concentrations of peptides such as IGF-I frequently fail to correlate with local tissue concentrations in CRC patients, rendering serum measurements an imprecise surrogate for the cellular processes occurring at the tumor site [29]. This dissociation extends to other components of the IGF system, as the presence of the loss of imprinting of IGF-II in tumor tissue does not necessarily correspond to alterations in its circulating levels [57]. Anatomical location introduces additional measurement variability, with the expression of signaling components proving significantly higher in rectal tumors compared to those arising in the ascending colon, suggesting that tumor location must be considered when interpreting biomarker measurements [29]. These observations indicate that systemic measurements may not adequately capture the paracrine signaling dynamics between tumor cells and stromal elements that operate at the tissue level [23]. Proteolytic fragments of IGFBPs generated by tumor-associated enzymes represent potential circulating surrogates of local ECM remodeling that could better reflect the local TME activity in systemic measurements, though their diagnostic utility in CRC requires prospective evaluation [80,187].

The success of biomarker validation depends critically on controlling pre-analytical variables that can introduce substantial bias into measured values. The choice of sample matrix proves essential, as biomarkers such as IGFBP-2 have been shown to yield higher and more consistent measurements in serum collected via separator tubes compared to plasma collected in EDTA or citrate anticoagulant tubes, indicating that the collection method itself can influence protein stability and detectability [255]. Storage conditions also exert significant effects on biomarker measurements, with some proteins demonstrating concentration changes under different storage protocols, while transcriptomic biomarkers prove particularly sensitive to temporal factors, with gene expression profiles in peripheral blood mononuclear cells exhibiting significant changes within two hours of blood withdrawal [257].

The clinical implementation of biomarkers for CRC necessitates the establishment of rigorously validated cutoff thresholds to ensure diagnostic accuracy across diverse patient populations. The determination of these thresholds requires the careful statistical consideration of sensitivity and specificity trade-offs, with different mathematical methods potentially producing distinct optimal values depending on the clinical context and the relative costs of false positives versus false negatives. A plasma IGFBP-2 cutoff of 377 ng/mL has been identified as providing a sensitivity of 80% and a specificity of 64% for CRC diagnosis in specific cohorts, demonstrating the feasibility of defining actionable thresholds [57], whereas other studies involving metastatic patients have reported median levels exceeding 613 ng/mL, which highlights how absolute biomarker concentrations can vary across different populations and disease stages [250]. Similarly, DNA methylation assays for IGF pathway genes have emerged as relevant quantitative biomarkers that detect epigenetic alterations contributing to IGF axis dysregulation in CRC. For example, these methylation assays are informative for detecting loss of imprinting (LOI) at the differentially methylated region, which leads to oncogenic IGF-II overexpression, as well as the promoter hypermethylation silencing of the protective IGFBP-3 gene [29,63]. These epigenetic alterations warrant standardized cutoffs to distinguish pathological methylation states from normal variation [29,63]. In quantitative DNA methylation assays, a percentage of methylated reference cutoff of four has been established to definitively correlate with protein silencing and differentiate methylation-positive colorectal tumors from negative ones [256]. Single biomarker cutoffs can vary by demographic, biological and methodological factors, including race, genetic composition, geographic region, and sample size, supporting the need for standardized thresholds for integrated multi-biomarker panels. The prospective testing of such panels in demographically varied cohorts may provide more robust and generalizable performances for precision medicine applications [252,258].

### 5.3. Clinical Translation Challenges

A primary challenge impeding clinical implementation lies in the scarcity of prospective validation studies required to demonstrate that candidate markers perform adequately under real-world screening conditions, particularly for the detection of precancerous lesions, such as advanced adenomas [259]. All biomarkers must undergo rigorous testing in prospective screening settings because performance estimates derived from retrospective case–control studies frequently prove inaccurate when applied to unselected populations, where the disease prevalence differs substantially from the enriched study cohorts typically used in discovery research [260]. Furthermore, clinical trial populations often lack demographic diversity, needing validation in specific groups to avoid disparities in diagnostic accuracy [252,261]. Although DNA methylation patterns and other molecular detection methods demonstrate promise as diagnostic indicators, most of these markers still require validation in large-scale prospective trials before they can be confidently integrated into routine clinical practice, representing a substantial gap between biomarker discovery and clinical utility [32,258].

This translational gap is particularly pronounced for IGF axis components in CRC. Despite decades of evidence linking circulating IGFBP-2, IGF-I/IGFBP-3 ratios, and IGF-II to CRC risk and prognosis, no IGF-derived biomarker has achieved United States Food and Drug Administration (FDA) approval for CRC screening or diagnosis. This reflects systemic challenges in cancer biomarker translation, as an estimated 0.1% of published cancer biomarkers ultimately enter clinical practice [262], a failure rate driven by underpowered discovery studies, reliance on retrospective case–control designs with enriched disease prevalence [263] and insufficient analytical standardization across laboratories [264].

Several factors specific to the IGF axis compound these general obstacles. First, CRC molecular heterogeneity means that IGF pathway activation varies substantially across CMS subtypes, with stromal and TGF-β-mediated immunosuppression most prominent in mesenchymal CMS4 tumors but less characterized in CMS1 and CMS3, limiting the generalizability of single-pathway biomarkers across the molecular spectrum [11,239]. Second, the functional decoupling between systemic circulating levels and local TME activity means that serum measurements may inadequately capture the paracrine signaling dynamics most relevant to tumor biology, as circulating and local concentrations of IGFs often do not correlate [29]. Third, metabolic comorbidities prevalent in CRC populations, particularly obesity, type 2 diabetes, and chronic hyperinsulinemia, independently alter circulating IGF and IGFBP levels, introducing confounding that reduces diagnostic specificity in the very populations at highest CRC risk [22,184]. Fourth, the proteolytic processing of IGFBPs by tumor-associated enzymes generates fragments with distinct immunoreactivities [168], and the current ELISA platforms vary in their ability to distinguish intact from cleaved forms, contributing to inter-assay discrepancies [255].

The regulatory landscape further constrains clinical adoption. The Centers for Medicare and Medicaid Services established minimum performance thresholds for blood-based CRC screening tests, requiring a sensitivity of at least 74% and a specificity of at least 90% compared to colonoscopy as the reference standard [265]. Current IGFBP-2-based panels, while demonstrating improved diagnostic accuracy over individual markers, have not yet met these benchmarks in recent case–control studies, highlighting the need for validation in prospective screening populations [251,252]. Notably, while large-scale interventional trials evaluating comprehensive IGF axis protein panels remain scarce, an ongoing prospective observational study is specifically assessing the diagnostic role of serum IGFBP-3 compared to carcinoembryonic antigen (CEA) in recently diagnosed CRC patients [266]. This absence of dedicated prospective evaluation for multi-marker panels represents a critical translational bottleneck. Future efforts should prioritize multi-institutional, prospective cohort studies that evaluate IGFBP-2-anchored multi-marker panels in demographically diverse screening populations, incorporating pre-specified cutoffs validated across different sample matrices and analytical platforms [252,255], to determine whether the mechanistically coherent IGF–immune signatures described in this review can achieve the performance characteristics required for clinical deployment.

Multivariate approaches for generating tumor-specific proteomic or multi-omics risk scores may substantially enhance the accuracy of prognostic predictions in long-term clinical settings, capturing aspects of tumor biology that single markers cannot adequately represent [254,267]. The demonstration that combining several plasma biomarkers improves overall diagnostic performance provides empirical support for integrated panels as more robust tools for identifying CRC at both early and advanced stages, with the additional potential to maintain performance across diverse populations where individual biomarkers may exhibit variable characteristics [252]. Given that IGF axis dysregulation and immune evasion operate through interconnected signaling networks in CRC, multi-marker signatures that capture both dimensions may provide a more biologically coherent assessment of tumor status than panels restricted to a single pathway.

The successful implementation of blood-based biomarkers requires evidence-based integration with established screening paradigms to maximize the population benefit while maintaining cost-effectiveness. Blood-based tests have been shown to demonstrate greater patient acceptability compared to stool-based screening options, addressing the common problem of fecal aversion that reduces adherence to fecal immunochemical testing, as well as the invasive nature of colonoscopy that creates barriers for many individuals [259]. The clinical success of novel non-invasive options, such as cell-free DNA tests, depends fundamentally on coordinating their deployment with appropriate screening intervals and ensuring sustained patient adherence through well-designed follow-up protocols [261]. Although blood-based tests do not permit immediate therapeutic interventions in the manner that colonoscopy does, their accessibility, affordability and non-invasive nature offer strategic advantages for improving screening adherence, particularly among populations with historically low participation rates [252]. The combination of traditional methods, such as fecal immunochemical testing, with lower gastrointestinal endoscopy continues to represent the most effective strategy for the accurate detection of colorectal neoplasia, suggesting that blood-based biomarkers may function optimally as complementary tools within tiered or risk-stratified screening algorithms that can accommodate varying levels of patient risk and preference [14,268]. The effectiveness of such tiered approaches may depend on capturing the systemic footprint of bidirectional IGF–immune crosstalk discussed throughout this review. Circulating IGF system components, particularly IGFBP-2, have already demonstrated improved diagnostic performances within multivariate blood panels [251,252,255], and their integration with peripheral immune signatures could yield rationale-based screening tools that reflect both growth factor dysregulation and mechanistically linked immunosuppressive remodeling in CRC.

## 6. Discussion

This review integrates the current evidence on bidirectional IGF–immune crosstalk that shapes CRC tumor phenotypes and therapeutic vulnerability. The IGF system does not merely promote tumor cell proliferation but actively orchestrates immunosuppression through coordinated effects on multiple immune cell populations, while inflammatory signals reciprocally reshape IGF bioavailability, creating self-reinforcing circuits that determine whether tumors remain immunologically “hot” or “cold”.

Three mechanistic principles emerge from this synthesis. First, IGFBP-2 functions as a central coordinator linking growth factor signaling to immune evasion, driving M2 macrophage polarization, Treg differentiation, and T cell suppression through pathways that operate across primary and metastatic sites. Second, the IGF axis is highly context-dependent, varying across molecular subtypes and disease stages. Third, local paracrine IGF production by tumor cells, CAFs, and TAMs increasingly dominates over systemic endocrine regulation as disease advances, which may explain the limited correlation between circulating IGF-I and TME activity.

Multi-marker panels incorporating IGFBP-2 alongside immune and metabolic biomarkers demonstrate superior diagnostic performances compared to individual markers, offering a path toward blood-based detection that could complement existing screening paradigms. The convergence of IGF signaling with checkpoint regulation, particularly the PI3K/AKT-mediated upregulation of PD-L1, suggests that the combined targeting of growth factor and immune pathways may overcome resistance mechanisms in MSS tumors that currently lack effective immunotherapy options.

This review offers several distinct contributions relative to the existing literature. Unlike prior reviews that have addressed the IGF system in CRC primarily from a growth factor signaling perspective [29,84], or that have examined the CRC immune microenvironment independently of growth factor networks [113,127], this work systematically maps the bidirectional interactions between IGF axis components and immune regulatory mechanisms. This integrative framework reveals interconnected feedback circuits, such as the IGFBP-2/STAT3/M2-TAM amplification loop [100,101] and the IGF-1R/PD-L1/CD8+ exhaustion axis [87,90,93,94], that are not apparent when either system is examined in isolation. Additionally, the synthesis across molecular subtypes highlights the disproportionate reliance of MSS and CMS4 tumors, characterized by high stromal infiltration and TGF-β activation, on paracrine IGF-mediated immunosuppression, providing a mechanistic rationale for the poor immunotherapy responses observed in these subtypes [11,47,239]. The integration of biomarker evidence with mechanistic pathways further bridges basic biology and clinical translation, an approach not systematically undertaken in previous IGF-focused reviews.

Several limitations should be acknowledged. First, much of the evidence connecting IGF signaling to specific immune cell phenotypes is derived from pan-cancer studies or non-CRC models, particularly for IGFBP-2/STAT3-driven M2 macrophage polarization (demonstrated in pancreatic cancer [100]), the IGFBP-3-mediated suppression of CD8+ T cell infiltration (demonstrated in breast cancer [105]), and the IGF-1R-dependent downregulation of antigen-processing machinery (demonstrated in prostate cancer [93]). Although the shared downstream signaling cascades (PI3K/AKT, MAPK, STAT3) support the plausibility of these mechanisms in CRC, direct experimental validation in CRC-specific models remains necessary, and human CRC studies simultaneously profiling IGF system components and immune populations across molecular subtypes are still scarce. Second, as a narrative review, the literature selection was guided by relevance to the bidirectional IGF–immune framework rather than by predefined search protocols, which may have introduced selection bias. Third, the immunomodulatory functions of individual IGFBPs beyond IGFBP-2 and -3 are poorly characterized in CRC, limiting the comprehensiveness of this synthesis. Fourth, the clinical biomarker evidence for IGF–immune signatures is predominantly retrospective. While several large prospective cohort studies have evaluated individual IGF axis proteins (such as IGF-1 and IGFBP-3) for CRC risk and early neoplasia [85,163,269], as well as one registered prospective trial [266], no prospective trials have specifically validated IGF axis protein panels or integrated IGF–immune signatures for early CRC detection. Finally, the potential contribution of IGF axis components to disease penetrance or immune surveillance in hereditary CRC syndromes remains unexplored. While isolated studies have reported associations between IGF1 gene polymorphisms and hereditary nonpolyposis CRC risk [270], the primary molecular drivers of familial adenomatous polyposis and Lynch syndrome operate through pathways mechanistically distinct from IGF signaling [29], and dedicated investigation is needed to determine whether IGF–immune interactions identified in sporadic CRC apply to these hereditary contexts.

To mitigate the risks of reporting and interpretation bias inherent to narrative reviews, several methodological safeguards were adopted during manuscript preparation. The relevant literature was identified through focused searches of PubMed, EBSCOhost, and Web of Science using combinations of IGF axis and immune-related terms, complemented by the backward citation tracking of key references. Inclusion prioritized peer-reviewed primary studies, with preference given to CRC-specific evidence; findings derived from non-CRC models were retained generally when they addressed unclear or underexplored mechanisms for CRC and are explicitly labeled as such throughout the text.

## 7. Conclusions

The bidirectional interactions between the IGF axis and immune regulation establish mutually reinforcing regulatory loops that shape CRC tumor phenotypes, progression, and therapeutic vulnerability. Integrated IGF–immune biomarker strategies offer a promising avenue for improving CRC detection and may complement existing screening approaches. Therapeutic strategies that combine targeting of growth factor signaling and immune regulation may help overcome resistance in MSS tumors that currently lack effective immunotherapy options. Prospective validation of integrated IGF–immune signatures in demographically diverse populations, together with mechanistic studies on immunocompetent CRC models, will determine whether the bidirectional interactions mapped in this review can be translated into clinical benefit.

## Figures and Tables

**Table 1:** The key components of the IGF axis and their roles in CRC immunity.

Component	Function	CRC Relevance	Immune Role	References
Ligands				
IGF-I	Primary mitogenic ligand; binds IGF-1R Activates PI3K/AKT and MAPK/ERK signaling cascades Stimulates cell proliferation, survival, and differentiation	Elevated IGF-I/IGFBP-3 ratio is associated with increased CRC risk Promotes tumor cell proliferation, survival, and angiogenesis in CRC	Promotes M2 macrophage polarization via AKT pathway Stimulates Treg proliferation but suppresses de novo Treg differentiation; favors Th17 differentiation via PI3K/AKT/mTOR Induces CD8+ T cell exhaustion and PD-1 upregulation upon chronic activation	[36,81,83,84,85,86,87,88,89]
IGF-II	Binds IGF-1R, IR-A, and IGF-2R Overexpressed via LOI; acts as a mitogen primarily through IR-A	Upregulated in CRC; drives autocrine and paracrine tumor growth CAF-derived IGF-II promotes invasion via YAP1 signaling	Induces tolerogenic DC phenotype through IL-10 secretion Promotes T cell exclusion via CXCL12/PD-L1 upregulation in stromal compartment Drives MDSC recruitment and expansion in IGF-II-high CRC Low-dose IGF-II/IGF-2R reprograms macrophages toward oxidative phosphorylation via GSK3	[23,24,63,88,90,91,92]
Insulin	Binds IR-A and IR-B; primary metabolic regulator Stimulates glucose uptake and lipid synthesis	Hyperinsulinemia suppresses IGFBP-1 and IGFBP-2, increasing free IGF bioavailability Enhances GLUT1 expression and aerobic glycolysis in tumor cells	Indirect immune role. Suppression of IGFBPs increases free IGF bioavailability, amplifying downstream immunosuppressive signaling in TME	[22,84]
Receptors				
IGF-1R	Primary mitogenic receptor with intrinsic tyrosine kinase activity Activates PI3K/AKT/mTOR and RAS/RAF/MEK/ERK signaling	Overexpressed in CRC; expression increases with Dukes stage Contributes to therapy resistance and undergoes nuclear translocation in advanced disease	Promotes M2 TAM polarization through AKT signaling Supports MDSC survival, recruitment, and immunosuppressive function Drives Treg expansion while suppressing de novo Treg differentiation Upregulates PD-L1 via STAT3 signaling; downregulates APM, including TAP1/2 and beta-2-microglobulin, in prostate cancer models Promotes RIG-I degradation, impairing innate immune sensing	[29,36,81,82,88,89,93,94]
IGF-2R	Scavenger receptor with no intrinsic kinase activity	Functions as a tumor suppressor gene; frequent LOH in CRC	Low-dose IGF-II/IGF-2R signaling reprograms macrophages toward an anti-inflammatory, oxidative phosphorylation-dependent phenotype via GSK3	[23,29,88,91,92]
IR-A	Binds both insulin and IGF-II; preferentially activates mitogenic pathways Heterodimerizes with IGF-1R to form hybrid receptors	Overexpressed in CRC relative to IR-B; hybrid IR-A/IGF-1R receptors expand receptor signaling capacity	IGF-II/IR-A association generates immunosuppressive macrophage phenotypes that promote angiogenesis, ECM remodeling, and immune evasion in breast cancer and other solid tumors	[22]
IR-B	Metabolic signaling isoform; predominantly expressed in liver, adipose tissue, and skeletal muscle	Relatively reduced compared to IR-A in CRC	Limited direct immune role; contributes to systemic metabolic regulation that indirectly influences IGF bioavailability	[22]
Binding Proteins				
IGFBP-1	Insulin-sensitive binding protein; sequestrates IGF-I PKCα-mediated phosphorylation increases its affinity for IGF-I under nutrient deprivation	Suppressed by hyperinsulinemia, increasing free IGF-I availability Nutrient deprivation and FOXO1 activation modulate availability in TME	Cleaved by neutrophil-derived serine proteases, releasing bioactive IGF into TME Reduced IGFBP-1 levels indirectly amplify immunosuppressive IGF signaling	[84,88,95,96]
IGFBP-2	Oncogenic binding protein with STAT3/NF-κB signaling activity Contains an RGD motif enabling integrin binding and IGF-independent signaling	Elevated in CRC; correlates with tumor burden, stage, and mortality. A key diagnostic and prognostic biomarker in CRC Subject to oxidative modifications by ROS in CRC patients Secreted by MAFs to suppress T cell activity Identified as a universal colon-specific SASP component	Induces M2 macrophage polarization via STAT3/IL-10 axis in pancreatic cancer models Drives Treg differentiation through STAT3 signaling Suppresses CD4+ and CD8+ T cell proliferation and CD25 expression in metastatic CRC via MAF-derived secretion Upregulates PD-L1 expression through EGFR/STAT3 in melanoma Participates in a positive feedback loop with HIF-1α under hypoxia	[57,88,97,98,99,100,101,102,103]
IGFBP-3	Most abundant circulating IGFBP; carries approximately 85% of IGFs in ternary complex with ALS Proapoptotic and growth-inhibitory in multiple contexts	Reduced expression in CRC relative to normal mucosa Silenced by promoter hypermethylation in CRC Subject to abnormal glycosylation in CRC patients	Negatively correlates with CD8+ T cell infiltration in breast cancer models, suggesting a suppressive role on T cell accumulation Induces CD38-high MDSCs with enhanced immunosuppressive capacity in murine tumor models	[29,88,104,105]
IGFBP-4	Inhibitory binding protein; PAPP-A-mediated proteolysis releases bound IGF Acts as a negative regulator of IGF bioavailability	Secretion reduced by IL-1β and IL-6 in CRC epithelial cells, paradoxically increasing local IGF availability Functions as a SASP component; propagates paracrine senescence via IGF-II/IGF-2R cascades	Cleaved by neutrophil-derived serine proteases, releasing bioactive IGF into TME IL-1β/IL-6-mediated reduction directly links tumor inflammation to enhanced IGF bioavailability	[24,88,95,106,107,108]
IGFBP-5	Context-dependent roles in growth promotion and inhibition depending on tumor type	Limited direct CRC-specific data SASP component; induced by IL-6/STAT3 in senescent fibroblasts, linking senescence to IGF remodeling in TME	Expression positively correlates with M2 macrophage infiltration and PD-L1 expression in glioma SASP-derived IGFBP-5 alters IGF bioavailability in senescent tumor microenvironments	[88,109,110]
IGFBP-6	Preferentially binds IGF-II; acts as a tumor suppressor in several cancers Expression is induced by hypoxia in endothelial cells	Reduced expression correlates with tumor cell proliferation, invasion, and poor survival in CRC	Exerts anti-angiogenic activity under hypoxic conditions, limiting tumor vascularization Expression is upregulated by IL-6, linking inflammatory signaling to IGF-II regulation	[29,49,88,111,112]

Abbreviations. ALS, acid-labile subunit; APM, antigen-processing machinery; CAF, cancer-associated fibroblast; CRC, colorectal cancer; DC, dendritic cell; ECM, extracellular matrix; GLUT1, glucose transporter 1; GSK3, glycogen synthase kinase 3; HIF-1α, hypoxia-inducible factor 1-alpha; IGF, insulin-like growth factor; IGFBP, IGF binding protein; IGF-1R/-2R, IGF receptor 1/2; IR-A/-B, insulin receptor isoform A/B; LOH, loss of heterozygosity; LOI, loss of imprinting; MAF, metastasis-associated fibroblast; MDSC, myeloid-derived suppressor cell; NF-κB, nuclear factor kappa B; PAPP-A, pregnancy-associated plasma protein A; PD-L1, programmed death-ligand 1; PI3K, phosphatidylinositol 3-kinase; PKCα, protein kinase C alpha; RIG-I, retinoic acid-inducible gene I; SASP, senescence-associated secretory phenotype; STAT3, signal transducer and activator of transcription 3; TAM, tumor-associated macrophage; TAP, transporter associated with antigen processing; TGF-β, transforming growth factor-beta; TME, tumor microenvironment; Treg, regulatory T cell; VEGF, vascular endothelial growth factor; YAP1, yes-associated protein 1.

## Data Availability

No new data were created or analyzed in this study.

## Abbreviations

The following abbreviations are used in this manuscript:

3′-UTR	3′-untranslated region
AKT	Protein kinase B
AMP	Adenosine monophosphate
AMPK	AMP-activated protein kinase
AP-1	Activator protein 1
APC	Adenomatous polyposis coli
APM	Antigen-processing machinery
Arg1	Arginase-1
ASCT2	Alanine-serine-cysteine transporter 2
ATP	Adenosine triphosphate
BAD	Bcl-2-associated death promoter
Bcl-2	B-cell lymphoma 2
Bcl-xL	B-cell lymphoma-extra large
CAFs	Cancer-associated fibroblasts
CEA	Carcinoembryonic antigen
CHI3L1	Chitinase-3-like protein 1
circRNA	Circular RNA
CMSs	Consensus molecular subtypes
CRC	Colorectal cancer
CTLA-4	Cytotoxic T-lymphocyte-associated antigen 4
CXCL	C-X-C motif chemokine ligand
CXCL12	Stromal cell-derived factor 1
DKK3	Dickkopf-related protein 3
ECM	Extracellular matrix
EGFR	Epidermal growth factor receptor
ELISA	Enzyme-linked immunosorbent assay
ERAP-1	Endoplasmic reticulum aminopeptidase-1
ERK	Extracellular signal-regulated kinase
FDA	U.S. Food and Drug Administration
FOXO	Forkhead box protein O
FOXP3	Forkhead box P3
GITR	Glucocorticoid-induced TNFR-related protein
GLUT1	Glucose transporter 1
GSK3	Glycogen synthase kinase 3
HIF-1α	Hypoxia-inducible factor 1-alpha
IFN-γ	Interferon-gamma
IGF-I/II	Insulin-like growth factor I/II
IGF-1R/-2R	Insulin-like growth factor receptor 1/2
IGFBP	Insulin-like growth factor binding protein
IGF2BP1	IGF-2 mRNA-binding protein
IL	Interleukin
iNOS	Inducible nitric oxide synthase
IR-A/-B	Insulin receptor A/B
IRS	Insulin receptor substrate
JAK	Janus kinase
lncRNA	Long non-coding RNA
LOI	Loss of imprinting
m6A	N6-methyladenosine
MAFs	Metastasis-associated fibroblasts
MAPK	Mitogen-activated protein kinase
MEK	Mitogen-activated protein kinase kinase
MHC	Major histocompatibility complex
miRNA	MicroRNA
MMPs	Matrix metalloproteinases
MMR	Mismatch repair
MDSCs	Myeloid-derived suppressor cells
MSI-H/-L	Microsatellite instability—high/low
MSS	Microsatellite stable
mTOR	Mammalian target of rapamycin
mTORC1/2	mTOR complex 1/2
ncRNA	Non-coding RNA
NF-κB	Nuclear factor kappa B
NK	Natural killer
PAPP-A	Pregnancy-associated plasma protein-A
PD-1	Programmed death-1
PD-L1	Programmed death-ligand 1
PDK1	Phosphoinositide-dependent kinase-1
PI3K	Phosphatidylinositol 3-kinase
PIP2/PIP3	Phosphatidylinositol 4,5-bisphosphate/Phosphatidylinositol 3,4,5-trisphosphate
PKCα	Protein kinase Cα
PKM2	Pyruvate kinase M2
RACK1	Receptor for activated C kinase 1
RAF	Rapidly accelerated fibrosarcoma
RAS	Rat sarcoma
RBP1	Retinol-binding protein 1
RNA	Ribonucleic acid
ROS	Reactive oxygen species
Shc	Src homology 2 domain containing
SLC38A2	Solute carrier family 38 member 2
SOCS3	Suppressor of cytokine signaling 3
SPP1	Secreted Phosphoprotein 1
STAT3	Signal transducer and activator of transcription 3
TAMs	Tumor-associated macrophages
TAP	Transporter associated with antigen processing
TCR	T cell receptor
TGF-β	Transforming growth factor-beta
Th1/Th17	T helper cell type 1/17
TILs	Tumor-infiltrating lymphocytes
TME	Tumor microenvironment
TNF-α	Tumor necrosis factor-alpha
Tregs	Regulatory T cells
TYK2	Tyrosine kinase 2
VEGF	Vascular endothelial growth factor
YAP1	Yes-associated protein 1
α2-M	α2-macroglobulin
